# In vitro antimicrobial and anticancer potentials of green synthesized luminescent carbon quantum dots derived from artichoke leaves

**DOI:** 10.1038/s41598-025-99841-9

**Published:** 2025-05-09

**Authors:** Eman Abo Diab, M. Ghali, M. M. Mosaad

**Affiliations:** 1 Physics Department, Faculty of Science, Kafrelsheikh University, Kafr El-Sheikh, 33516 Egypt; 2 Institute of Basic and Applied Sciences, Egypt-Japan University of Science and Technology, New Borg Al-Arab, Alexandria, 21934 Egypt

**Keywords:** Carbon quantum dots (CQDs), Artichoke, Green synthesis, Antimicrobial activity, Materials science, Nanoscience and technology, Physics

## Abstract

Naturally derived carbon quantum dots (CQDs) are novel carbon-based nanomaterials with excellent traits. It is highly demanded to develop CQDs from biowaste that have excellent photostability, a simple synthesis approach, and an appealing output so that they can be used widely in various fields. Herein, highly fluorescent CQDs were synthesized hydrothermally using artichoke leaves. The CQDs were synthesized and analyzed for their structure, optical properties, antimicrobial, and anticancer activities. The CQDs exhibited antimicrobial action against a single fungus strain in addition to Gram-positive and Gram-negative cells; also, the cytotoxicity against the MCF-7 cell line was evaluated as 96.5 µg/mL. The findings indicated that the spherical dots have a semi-spherical shape with the smallest size of 2.88 nm, and a zeta potential value of 37.31 V, thus confirming that the synthetic CQDs are in an outstanding colloidal state. When photoexcited at 320 nm, the dots were found to show blue fluorescence at 398 nm with a fluorescence quantum yield of 3.32%, long fluorescence decay time, high photostability, and good sensing for hydrogen peroxide. Additionally, the effect of ionic strength was evaluated.

**Supplementary Information:**

The online version contains supplementary material available at 10.1038/s41598-025-99841-9.

## Introduction

Zero-dimensional carbon nanomaterials referred to as carbon quantum dots (CQDs) are usually smaller than 10 nm^1,2^. These materials are composed of the hybridized sp^2^/sp^3^ carbon atoms and have different surface functional groups^3^. Extensive research has been conducted to prepare and characterize various carbon-based nanomaterial systems, especially CQDs^1,3–13^due to their potential use as fluorescent probes. Among their attributes are their great luminosity intensity^14^, strong photostability, low toxicity^1,10^, small size, chemical inertness, biocompatibility, and enhanced electron transfer capacity over other nanomaterials and organic sensors^14^. They are extensively utilized in many areas such as optoelectronics, biomedical imaging^2,15^, pharmaceutical delivery, and water treatment^9^. Several potential sources have been explored in the development of these luminescent nanomaterials. These sources include chemical sources such as citric acid and urea^14,16–18^, glucose^3,10^, mandelic acid, ethylenediamine^12^ and polyacrylamide, as well as organic sources like sugars^19^, corn stalk shell^2^, wheat straw and bamboo residues^20^, banana peel waste^1^, watermelon juice^11^, agro waste^21^, tangerine juice^13^, ginkgo leaves^22^, apple juice^23^, P. acidus fruit juice^15^, grapefruit juice^8^, Orange peel^7^, and Lemon Juice^4^. The most dependable precursors for producing high-quality CQDs with good quantum yield are usually chosen as organic compounds. However, the widespread use of these precursors of aromatic hydrocarbons is severely limited by their toxicity^1^. To change the surface chemistry of CQDs with numerous functional groups, the choice of precursors is essential. This permits cellular imaging in complex biological samples and the sensing of specific analytes^23^. Natural materials contain various heteroatoms, resulting in CQDs with unique properties and surface groups even without further passivation or modification^1^. Moreover, it does not require using pricey or dangerous chemicals or difficult post-treatment procedures^23^.

Two approaches have been reported for synthesizing CQDs: top-down and bottom-up approaches^10,11,18^. Due to their expensive machinery and labor-intensive procedures, most top-down systems have manufacturing challenges and high production costs. Conversely, as compared to other techniques, bottom-up approaches provide a more straightforward and ecologically favorable option. Top-down methods comprise laser ablation, arc discharge, and electrochemical oxidation; bottom-up methods include solvothermal, hydrothermal, microwave, and ultrasonic treatments^1,18^. The hydrothermal process has garnered interest due to its ease of use, affordability, environmental friendliness, low temperature, economic advantages, and mass-producibility, all achievable in a one-pot process among the reported bottom-up methods^1^. Hydrothermal methods with a green chemistry nature have received extensive media coverage and employ natural predecessors to create CQDs^1,20^. Due to increased food demand, most food waste is disposed of by burning, burying, or feeding it to animals which wastes biological resources and causes pollution^2^. Combining hydrothermal processes with natural sources such as bio-waste and plant materials can offer advantages like reducing production costs, simplifying strategies, utilizing easily available raw materials, and minimizing toxicity^15^. Thus, synthesizing carbon-based nanomaterials can be an efficient method for making use of agricultural wastes^2^. It should be mentioned that the structure and chemical characteristics of CQDs created by hydrothermal treatment are mostly determined by the different precursors, which have different amounts of carbon atoms, chain lengths, and functional groups^3^.

**Scheme 1:**
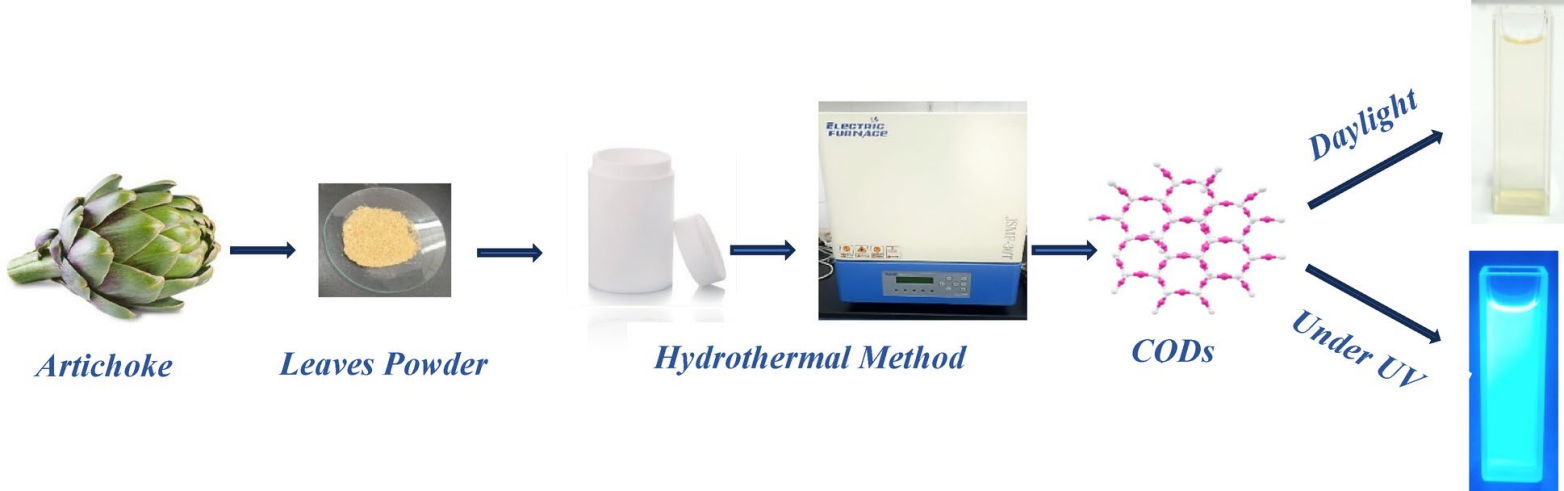
Procedure for synthesizing the fluorescent CQDs from the artichoke leaves.

In this work, we used artichoke (Cynara) leaves as an initial for synthesizing CQDs. Cynara is a tiny genus that comes from the Mediterranean region. It has two crops: the cardoon (*Cynara cardunculus* var. altilis DC) and the globe artichoke (*Cynara cardunculus* var. scolymus L.). *Cynara scolymus* (Asteraceae) is a local Mediterranean plant, also known as artichoke in Brazil. It is cultivated globally for its nutritional and medicinal properties^24^. The globe artichoke is grown primarily for its big, fleshy head, which is the plant’s immature flower and makes up between 30% and 40% of the fresh weight^24,25^. The receptacle, often called the “heart,” and the fragile inner leaves, or bracts, are the edible portions that make up 35–55% of the head’s current weight. Industrial by-products used in non-food applications, such as exterior bracts, leaves, and stems, account for approximately 80% of biomass and are rich in flavones and caffeoylquinic acids^26^ and could potentially be used for extracting food additives and nutraceuticals^24^. Thus, we are studying the degradation of artichoke leaves to produce high-value CQDs using the hydrothermal method, which has gained attention for its green and uncomplicated process (Scheme 1). Different analyses were used to approve the synthesis of CQDs, like Transmission Electron Microscope (TEM), Fourier transform infrared (FTIR) spectroscopy, Raman, Zeta potential, UV–Vis, steady state fluorescence, and fluorescence lifetime spectroscopy. Small nano-sized CQDs have been obtained and found to emit bright blue fluorescence under UV lamps. Also, under different excitation wavelengths (λex), the synthesized CQDs showed excitation-dependent fluorescence, water solubility, and strong resistance to photobleaching. Furthermore, the produced CQDs exhibited strong antibacterial activity and antitumor action.

## Experimental

### Materials

Artichoke was obtained from the local marketplace of Alex, Egypt, and employed as a precursor—deionized water was utilized for sample synthesis—isopropanol, ethanol, and EDTA. Artichoke leaves were cut, cleaned well, washed with distilled water, kept under the sun for 2 days to dry, and ground to powder. Mammalian cell lines: The American Type Culture Collection (ATCC, Rockville, MD) provided the human breast cancer cell line MCF-7. Essential chemicals: Sigma (St. Louis, Mo., USA) supplied the MTT and trypan blue dye; Lonza (Belgium) provided the fetal bovine serum, RPMI-1640, HEPES buffer solution, L-glutamine, gentamycin, and 0.25% trypsin-EDTA.

### Synthesis of carbon quantum dots

Artichoke leaves were used to synthesize the fluorescent CQDs using a hydrothermal process. Briefly, 0.4 g of fine artichoke leaves powder was incorporated with 50 mL of deionized water. Stir the mixture for 4 h and then sonicate for 1 h. A 50 mL stainless-steel autoclave with a Teflon lining was filled with just 35 mL of this solution, after which it was maintained at 240 °C for 18 h in a hot air oven. It was permitted to cool naturally to ambient temperature following the reaction. The resulting crude product, which was brownish yellow, was filtered using a syringe filter (0.22 µ) to eliminate the large-sized particles. Ultimately, the CQDs were collected via a dialysis membrane (1000 MWCO) for 72 h against deionized water. The obtained CQDs solution was kept at 4 °C until used ^10,15^. Sometimes it was necessary to prepare it as a solid to meet characterization requirements; in that case, the sample was made as follows: A portion of the CQDs solution was vacuum-freeze-dried for 3 days to produce a whole brown powder product that could be used for additional characteristics and applications.

**Scheme 2:**
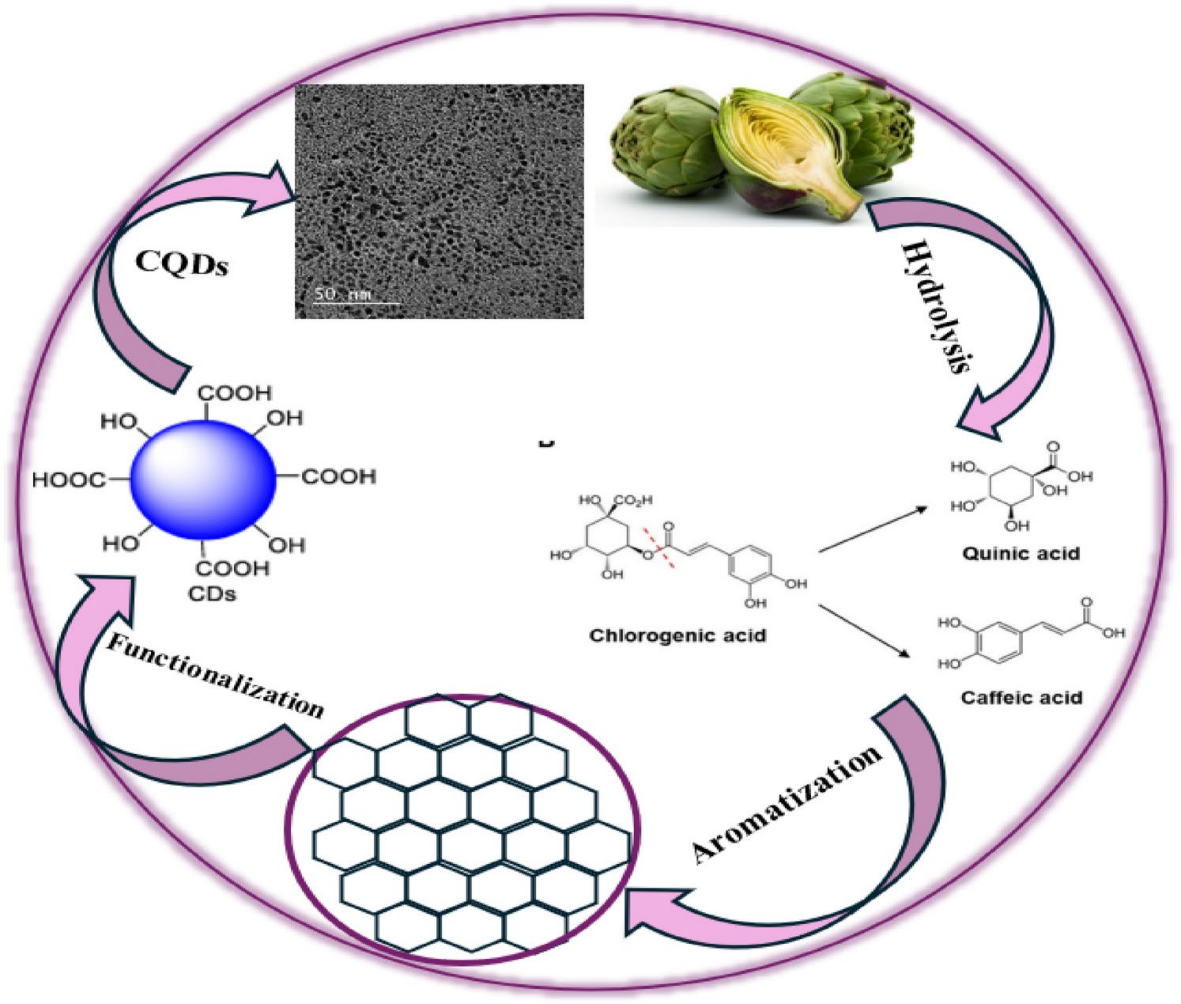
Schematic illustration of the mechanism of CQDs formation from artichoke leaves.

The leaves of artichokes are abundant in polyphenols, including chlorogenic acid (CGA), caffeoylquinic acids, and caffeic acid, as well as flavonoids, proteins/enzymes, and polysaccharides^27,28^. The suggested process for CQDs formation includes: (a) Initially, the breakdown of precursors occurs through the decomposition of polyphenols, with chlorogenic acid (CGA) breaking down through hydrolysis into caffeic acid and quinic acid^29^. (b) A subsequent thermal decomposition generates aromatic fragments and carboxyl groups. (c) Enzyme denaturation, particularly of cardosins, breaks them down into amino acids, which provide nitrogen for CQDs self-doping. (d) Subsequently, carbonization and nucleation happen through dehydration and condensation, with aromatic clusters from polyphenols and carbonized polysaccharides forming sp²-hybridized carbon cores. The amorphous core containing aromatic regions appears to be a transitional phase in the creation of CQDs, preceding the process of graphitization that leads to a structured crystal lattice^30^. The presence of oxygen- and nitrogen-containing functional groups (–OH, –COOH, –C-N-C) from leftover polyphenols and proteins helps to passivate the surface of CQDs, which improves both their solubility and fluorescence^28^ Scheme 2 summarizes these processes.

### In vitro cytotoxicity and antimicrobial activities examination

#### In vitro cytotoxicity of CQDs against MCF-7 cell line

The cytotoxic effect of CQDs against mammalian cell lines was assessed against MCF-7 HTB-22 cells (refer to a cell line derived from human breast cancer) using the MTT assay, which indicates the concentration needed to induce toxic effects observed in 50% of surviving cells (IC_50_). Cells were cultivated in RPMI-1640 medium with 50 µg/mL gentamycin and 10% inactivated fetal calf serum and subcultured two to three times per week. For the antitumor assay, MCF-7 cells were suspended in a medium at a concentration of 1 × 10^5^ cells/well in 96-well plates and incubated for 24 h. The CQDs were dissolved in PBS and then distributed into 96-well plates in triplicate, resulting in eight different concentrations of CQDs ranging from 7.8 to 1000 µg/mL. A control experiment with the media was conducted using a 96-well plate. After a period of incubation lasting 48 h, the viability of the cells was assessed using the MTT assay. From each well, 85 µl of media was removed, followed by the incorporation of 50 µL of DMSO, which was mixed and incubated for 10 min at 37 °C^31^. The optical density was subsequently assessed at 590 nm using a microplate reader (SunRise, TECAN, Inc, USA) to evaluate the count of living cells, and the viability percentage was computed as:

$$\:cell\:viability\:\left(\%\right)=\:\frac{OD\:\text{treated}}{OD\:control}\:100,$$

where OD treated refers to the mean optical density of wells that have been treated with the sample being tested, and OD control signifies the average optical density of the untreated cells^10^. The IC_50_ was determined by analyzing graphic representations of the dose-response curve for each concentration, utilizing GraphPad Prism software based in San Diego, California, USA. A nonlinear regression model that was specially designed to capture the sigmoidal connection between drug concentration and cellular response was used in the investigation. A four-parameter logistic (4PL) curve, which is a common method for figuring out important parameters like the IC_50_, could be precisely fitted with GraphPad Prism. This model takes into consideration: HillSlope: The steepness of the curve; Top: The maximum response value; Bottom: The minimum response value; IC_50_: The concentration at which the response is midway between the minimum and maximum values, also known as the inflection point.

#### In vitro antimicrobial potential examination

The disk diffusion approach was used to identify the CQDs’ inhibitory zone. The antimicrobial activities against five types of microorganism strains employing two Gram-positive bacteria (*Methicillin-resistant Staphylococcus aureus* (ATCC 33591) (*MRSA)* and *Staphylococcus aureus* (ATCC 6538)), two Gram-negative bacteria (*Klebsiella pneumoniae* (ATCC 13883) and *Escherichia coli* (ATCC 8739)), and one yeast fungus (*Candida albicans* (ATCC 10221)) have been used. These bacterial strains, especially *S. aureus* and *E. coli*, are frequently employed as model organisms in antibacterial testing because of their wide range of traits and clinical significance. Briefly, Mueller Hinton agar was used to prepare the agar plates for the bacterium isolates, and Sabouraud dextrose agar was used to prepare the fungus isolates. A sterile saline solution was used to suspend isolated colonies, and the turbidity was changed to (1.5 × 10^8^ CFU/mL) to provide standardized inoculants. Then, 50 µL of the diluted standard inoculants were evenly streaked onto the surface of the respective sterilized agar plate. A 50 µL bacterial culture solution at 1000 µg/ml was used to inoculate these plates. Each was put into a 96-well cell culture plate with a 100 µL final volume for each well and spread evenly across the surface. After 20 h of incubation at 37 °C, a digital vernier caliper was used to measure the inhibition zones surrounding the discs; zone diameters represented the level of antibacterial activity^32,33^.

#### MIC test

The minimum inhibitory concentration (MIC) value, like the bacterial growth inhibition zone in the qualitative method, is fundamental for evaluating the susceptibility or resistance of a pathogen to a specific antibiotic. In certain cases, determining the MIC is the only dependable phenotypic method for assessing drug sensitivity, especially when qualitative methods have yielded inaccurate results^34^. The minimal concentration of the substances that inhibited the development of microorganisms was identified as MIC^35^. It was executed on the same microorganisms (*S. aureus* (ATCC 6538), *MRSA* (ATCC 33591), *E. coli* (ATCC 8739), *K. pneumoniae* (ATCC 13883), and *C. albicans* (ATCC 10221)) as the antimicrobial activity test.

The inoculum preparation was done via the direct colony suspension method. This involves creating a broth or saline suspension from isolated colonies on a non-selective medium and then adjusting the turbidity to match a 0.5 McFarland standard. This standardization ensures a consistent 1 × 10^8^ to 2 × 10^8^ CFU/mL concentration. After standardization, we dilute the suspension to achieve approximately 5 × 10^5^ CFU/mL per well in microdilution trays. After 15 min of preparation, inoculate each well using a tool that provides ≤ 10% of the well volume. To ensure even temperature distribution, incubate the inoculated trays in an ambient air incubator for 20 h at 37 °C. By keeping trays from drying out, we created the ideal environment for precise susceptibility testing. The microorganisms were then grown in a Luria–Bertani (LB) liquid medium at 37 °C that enabled robust bacterial growth while being shaken overnight at 180 rpm. Through this technique, microorganisms were able to achieve a high density, guaranteeing enough biomass for further analysis. The microorganisms were then diluted to a concentration of approximately 1 × 10^6^ CFU/mL to make sure antimicrobial testing yields accurate and consistent results. The CQDs sample was prepared at varying concentrations using sequential twofold dilutions (e.g., 1000, 500, 250, 125, 62.5, µg/mL). Microorganisms suspended in an LB medium without CQDs served as the control, while the blank was solely LB medium. After that, the combinations were incubated for 20 h at 37 °C, enabling a comprehensive evaluation of CQDs’ antibacterial efficacy against microorganisms. Then, using a BioTek 800 TS microplate reader, the concentration of bacteria was ascertained by measuring the optical density at 600 nm (OD600)^32,36^. Typically, the concentration at which the OD600 value is significantly lower than the untreated control is used to calculate the MIC.

### Fluorescence quantum yield measurement

CQDs’ fluorescence quantum yield (QY) was determined by comparing the absorption values and fluorescence intensities of CQDs samples with those of quinine sulfate. To reduce the impact of reabsorption, the absorbance of the CQDs samples was maintained below 0.05 at the excitation wavelength^12^. As a standard sample, 0.1 M H_2_SO_4_ (η = 1.33) was used to dissolve quinine sulfate (QY = 54%), which was selected as the reference. On the other hand, CQDs were dissolved in DI water (η = 1.33) and excited at 320 nm. The following formula was used to determine the QY of CQDs^23^

$$\:{Q}_{CQDs}={Q}_{R}.\:\frac{{I}_{CQDs}}{{I}_{R}}.\:\frac{{A}_{R}}{{A}_{CQDs}}.\frac{{\eta\:}_{CQDs}^{2}}{{\eta\:}_{R}^{2}}$$

Q stands for fluorescence quantum yield, I is for integrated fluorescence intensity, A refers to the absorbance measured at the optimum excitation wavelength, and the index of refraction of the solvents utilized is indicated by η. The carbon dots are indicated by “CQDs”, while the reference sample is indicated by the subscript “R”. The fluorescence quantum yield was calculated to be 3.32 ± 0.046% at λex 320 nm.

### Characterization techniques

Structure characterization was made using TEM (FEG TEM (JEM-2100) − 200 kV). The Shimadzu diffractometer (XRD-6000) utilized Cu Kα1 radiation with a λ of 0.154 nm for structural analysis. Fourier transform infrared spectroscopy (FT-IR-6800, Jasco) measured the functional groups of CQDs over the range of 400–4000 cm^−1^. The Raman spectrum was evaluated by a (WITec alpha300 R Wissenschaftliche Instruments, Germany) WITec setup with two laser sources (532 nm, Max.30 mW, and 785 nm, Max.133 mW), Raman spectrometer with 785 nm laser was used as an excitation source. XPS measurements were performed using the K-ALPHA (Thermo Fisher Scientific, USA) with monochromatic Al K-alpha X-ray radiation ranging from 10 to 1350 eV and a spot size of 400 micrometers at a pressure of 10^− 9^ mbar, utilizing a full spectrum pass energy of 200 eV and a narrow spectrum pass energy of 50 eV. Using the Zeta potential analyzer Brookhaven instrument (Corp. Ver. 5.59, USA), the colloidal stability of the synthesized CQDs was tested. For optical characterization, a Shimadzu (2600 UV–Vis–NIR JAPAN) spectrophotometer was utilized to conduct absorption spectra of a CQDs solution in the 200–800 nm wavelength range. A fluorescence spectrophotometer (JASCO FP-8300 s-USA) was used to perform each fluorescence spectrum.

## Results and discussion

### Carbon quantum dot synthesis conditions

**Fig. 1:**
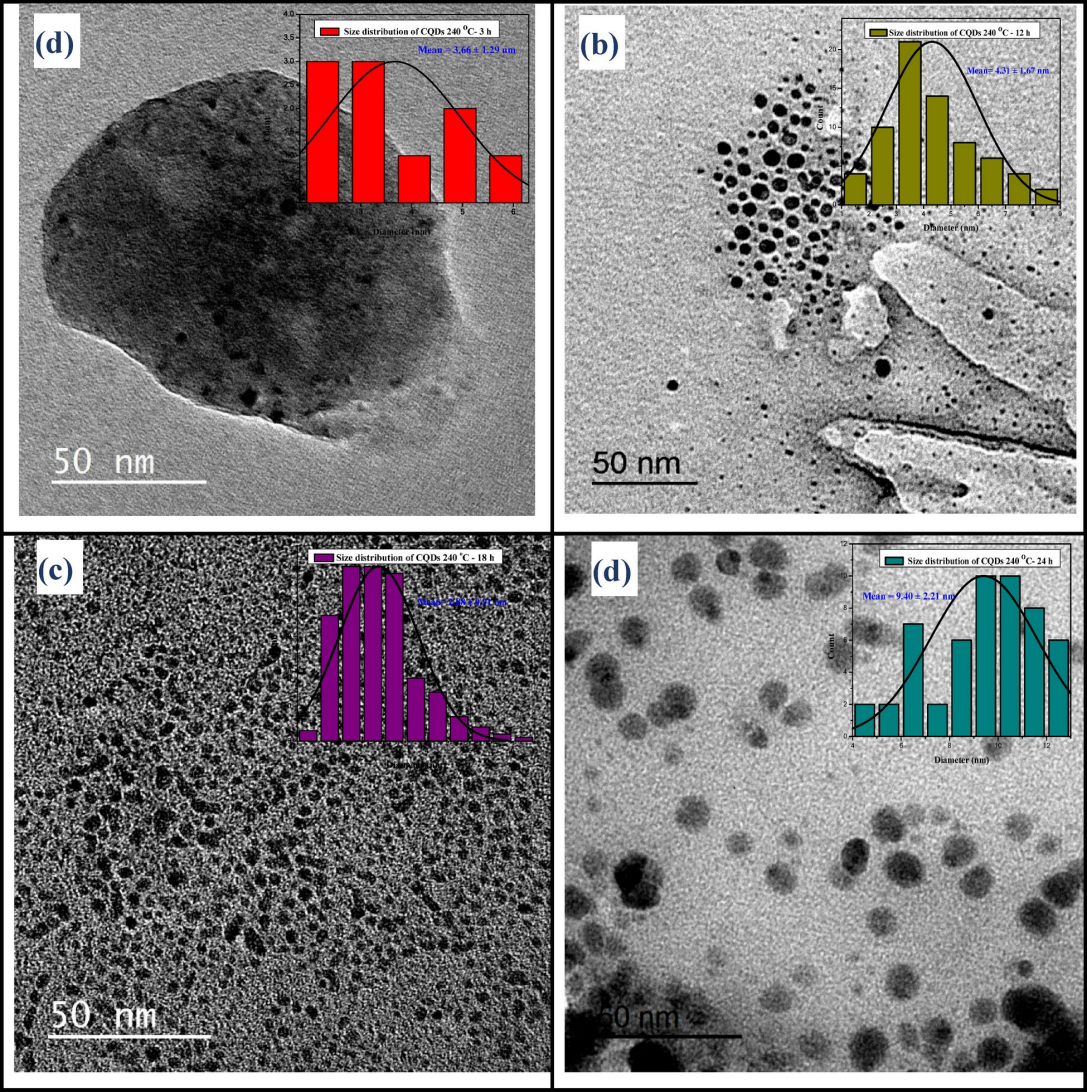
TEM image and inset: Size distribution of TEM image of CQDs (**a**) S1, (**b**) S2, (**c**) S3, and (**d**) S4 at 50 nm.

**Fig. 2:**
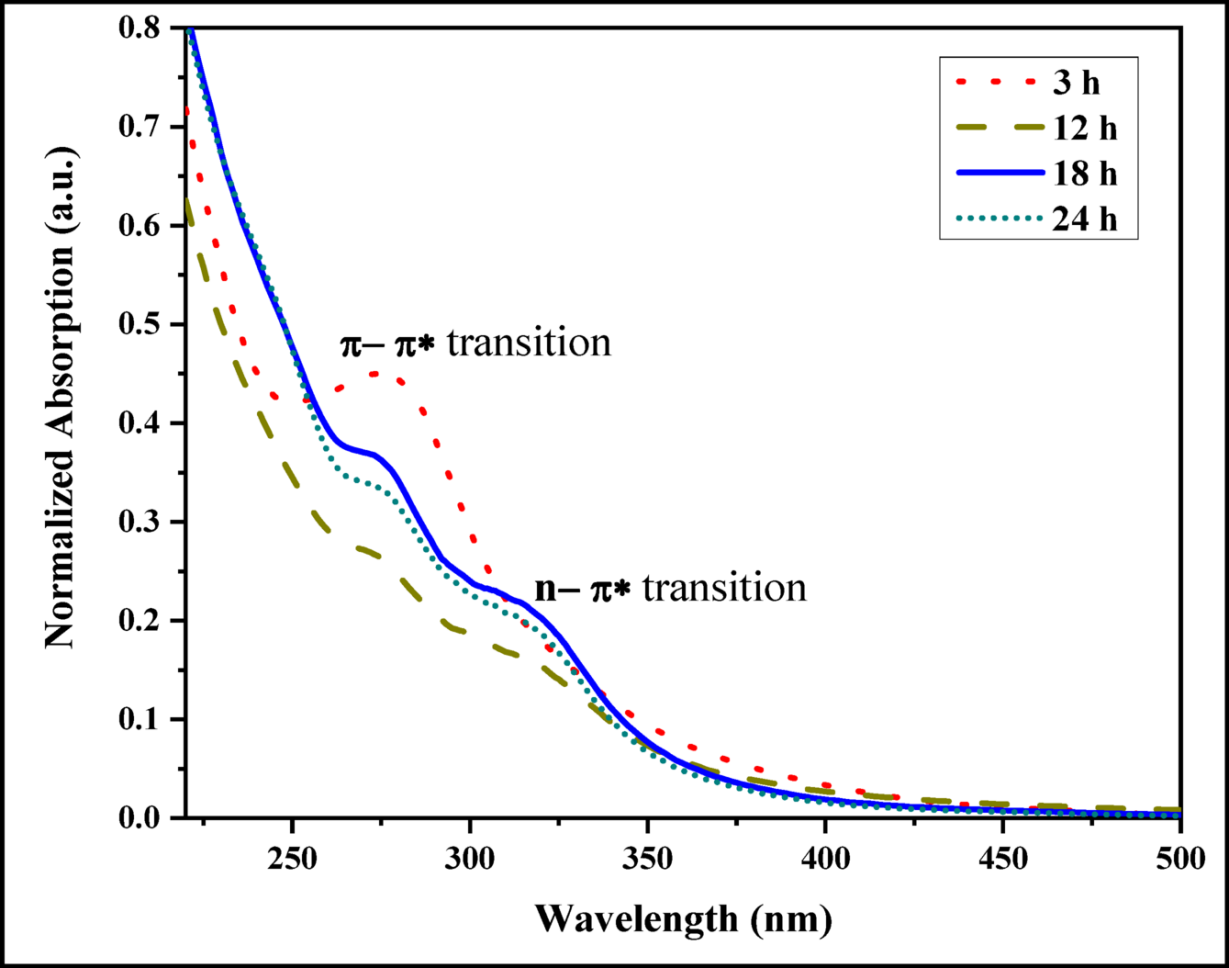
Normalized UV-Vis absorption of CQDs at various times.

**Fig. 3:**
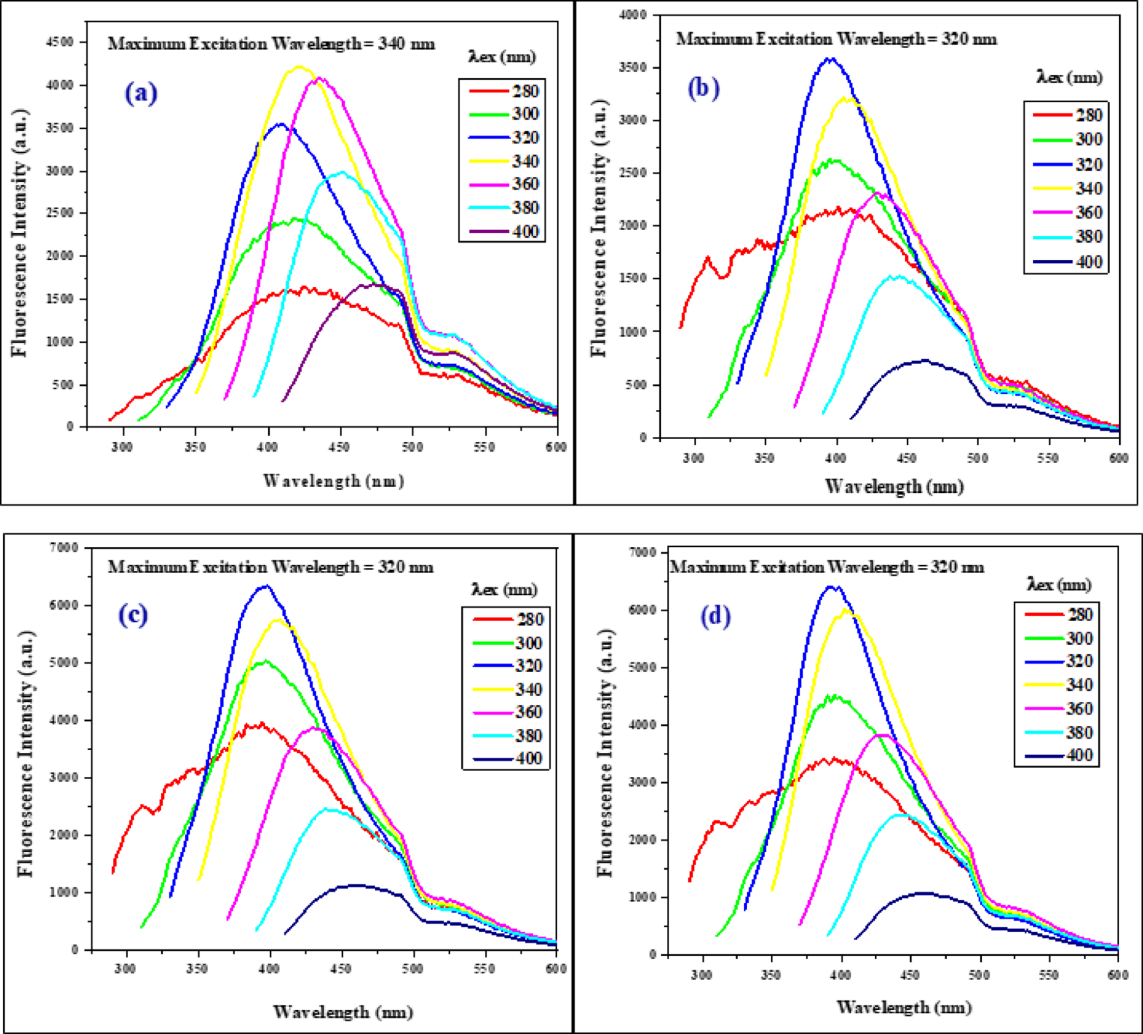
Excitation-dependent fluorescence emission spectra of CQDs (**a**) S1, (**b**) S2, (**c**) S3, and (**d**) S4.

Extensive research has pointed out that the fluorescence characteristics of CQDs are significantly influenced by technical manufacturing. To maximize the fluorescence performance of CQDs, a variety of reaction parameters, including reaction time and temperature, were examined. The reaction time was first examined with fixed concentration and temperature as synthesis conditions and compared based on TEM, UV, and PL. The results of which are shown in Figs. 1, 2 and 3. Briefly, four samples at different reaction times have been synthesized and identified as S1 = 3 h, S2 = 12 h, S3 = 18 h, and S4 = 24 h. Upon analyzing TEM images, Fig. 1, it was found that the CQDs had a spherical shape with average sizes of 3.66 ± 1.29, 4.31 ± 1.67, 2.88 ± 91, and 9.41± 2.21 nm for S1, S2, S3, and S4, respectively.

For sample S1, Fig. 1a, it is noticed that a few dots can be hardly observed, most likely due to the short reaction time, where the dots did not have sufficient time to disperse throughout the sample. By increasing the reaction time as in S2, S3, and S4 in Fig. 1b, the CQDs could be distributed evenly throughout the sample, so dispersed particles can be easily observed in the TEM images. Based on UV-Vis results in Fig. 2, the C = O bond’s n–π* transition and the C = C bond’s π–π* transition is detected at 317 nm and 277 nm, respectively, which are characteristic of the chromophores present in the CQDs. These transitions are driven by the functionalities found on the surface of CQDs^9^.

Using gradually longer excitation wavelengths (from 280 to 400 nm), the PL emission spectra were examined. Photoluminescence’s result proved that as the wavelength of the excitation increases, the emission peak moves to a higher wavelength as shown in Fig. 3. Hence, the dots show excitation-dependent emission characteristics and emit maximum emission fluorescence at λex 340, 320, 320, and 320 nm for S1, S2, S3, and S4, respectively. The probabilities of the surface transition modes vary at different excitation wavelengths because of the different functional groups that are visible on CQD surfaces. A change in the emission spectra results from each transition dominating at the appropriate excitation wavelength^23^. Therefore, when the excitation wavelength increases, the intensity of the emission progressively rises to its maximum at Ex 320 nm—the highest intensity of emissions is attained at 398 nm - and then constantly falls; this indicates the ability to tune the emission wavelength of CQDs. The emission of the CQDs showed a clear excitation-dependent performance, in line with other research^1,2,22,23,37^ see Table 1.

**Scheme 3:**
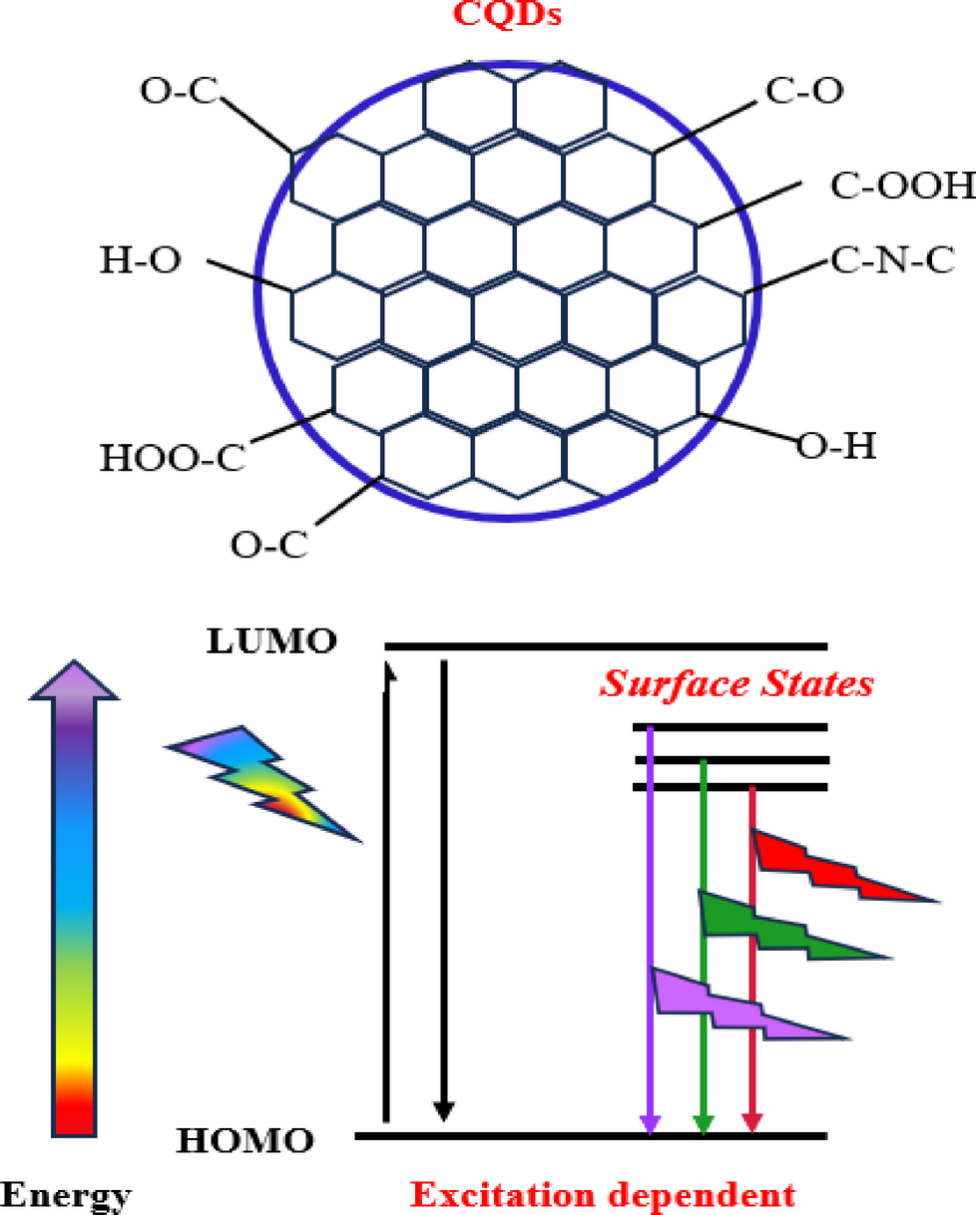
The role of functional groups in introducing surface states on CQDs surface.

Fluorescence can arise from quantum confinement effects, defect states, molecular fluorophore states, and cross-chain enhanced emission states^38^. Here, the photoluminescent behavior of carbon dots is mainly explained by emissive traps on the dots’ surface^9^. Additionally, there were differences in energy levels (i.e., surface states) depending on the existence of distinct functional groups like C–O, C=O, and O=C–OH, which significantly affect the emission properties of CQDs^23^ see Scheme 3. It is important to note that, for all studied samples, the maximum intensity PL peak position does not change with the dots’ average size, which indicates that the quantum size effect is not the dominant mechanism of fluorescence. Also, we found that sample S3 shows the highest luminescence intensity. This can be interpreted that smaller CQDs can have a higher surface-to-volume ratio, which might lead to more surface defects that can enhance fluorescence intensity. The optimal size for photoluminescence can vary based on the synthesis conditions and the specific surface functionalities present. So, 18 h was determined to be the ideal time for synthesis.

**Table 1:** The optimum excitation-emission wavelength, average size of the particles, and fluorescence quantum yield for some previous works.

Precursor	Nanomaterial	Optimum excitation wavelength	Emission wavelength	Average particle size	Quantum yield	References
Polyacrylamide	Nitrogen-doped carbon quantum dots (N-CQDs)	360 nm	455 nm	3 nm	23.1%	^18^
Mandelic acid and ethylenediamine	Nitrogen-doped carbon dots (N-CDs)	342 nm	429 nm	2.5 nm	41.4%	^12^
Watermelon juice	Nitrogen-doped carbon quantum dots (N-CQDs)	355 nm	439 nm	3–7 nm	10.6%	^11^
Glucose and m-phenylenediamine	Nitrogen-doped carbon quantum dots (N-CDs)	320 nm	460 nm	8 nm.	17.5%	^10^
Pomegranate peels	Carbon dots (CDs)	360 nm	440 nm	4.7 nm	0.1%	^21^
Ginkgo leaves	Carbon quantum dots (CQDs)	380 nm	442 nm	4.90 nm	11%	^22^
Apple juice	Carbon dots (CDs)	368 nm	475 nm	4.5 ± 1.0 nm	4.27%	^23^
Banana peel waste	Carbon dots (CDs)	355 nm	429 nm	5 nm	20%	^1^
Tangerine juice	Carbon Quantum Dots (CQDs)	–	–	1.1–2.6 nm	3.1%	^13^
Corn stalk shell	Carbon quantum dots (CQDs)	365 nm	460 nm	1.2–3.2 nm	16%	^2^
Citric acid and urea	Carbon quantum dots (CQDs)	380	453	2.57 nm	97.3 ± 0.5%	^16^
Artichoke leaves	Carbon quantum dots (CQDs)	320 nm	398 nm	2.8 ± 0.9 nm	3.32%	This work

**Fig. 4:**
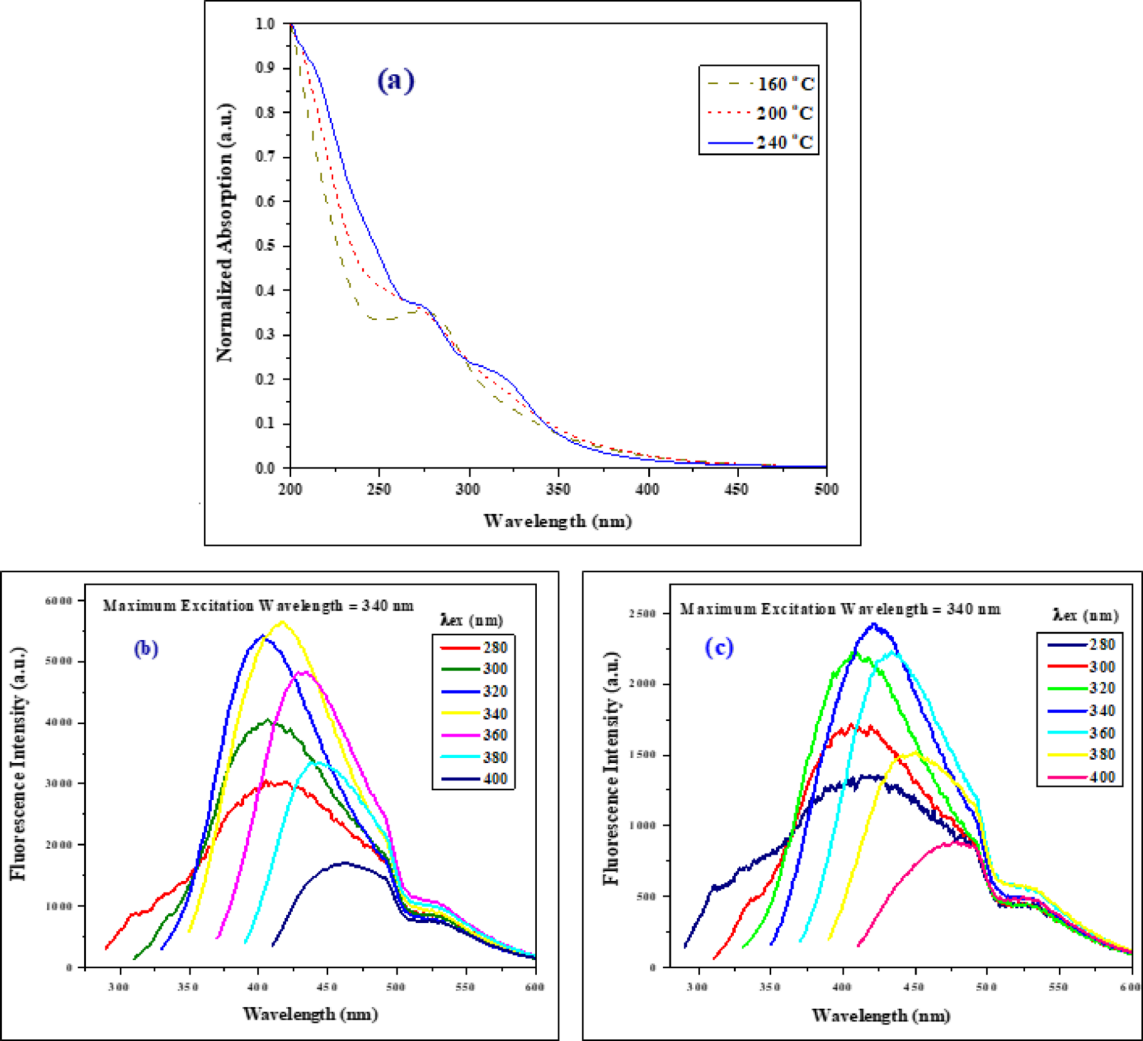
(**a**) Normalized UV–Vis absorption of CQDs at three different temperatures. CQDs’ emission spectra synthesized at (**b**) 200 °C and (**c**) 160 °C.

In addition, three temperatures were selected to ascertain the ideal circumstances for synthesis. Three were synthesized, the first at 240 °C, the second at 200 °C, and the last at 160 °C with a fixed concentration and 18 h as synthesis duration. Based on the results of the optical absorption, Fig. 4a, and fluorescence intensity, it was discovered that the sample prepared at 240 °C had a higher fluorescence intensity than those at 200 °C and 160 °C; see Figs. 3c and 4b,c. Because 240 °C was determined to be the ideal temperature, we will focus on discussing a sample that was prepared at 240 °C for eighteen hours in the discussion that follows.

### Characterization of the selected sample

#### Structural analysis

**Fig. 5:**
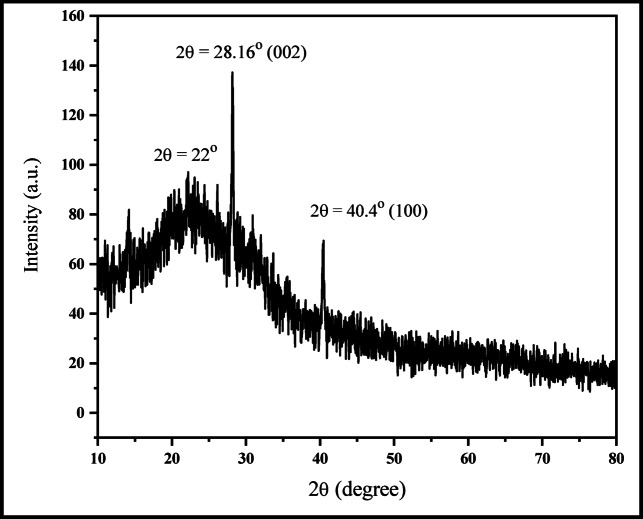
XRD analysis for CQDs.

The XRD pattern of CQDs showed a broad peak at about 2θ = 22^O^, revealing an amorphous carbon phase^1^. The sharp peaks at 28.16º and 40.40º correspond to the (002) dispersions^23,39^ and (100)^39^,

indicating the presence of graphitic carbon in the CQDs (Fig. 5).

#### Morphological and surface analysis

**Fig. 6:**
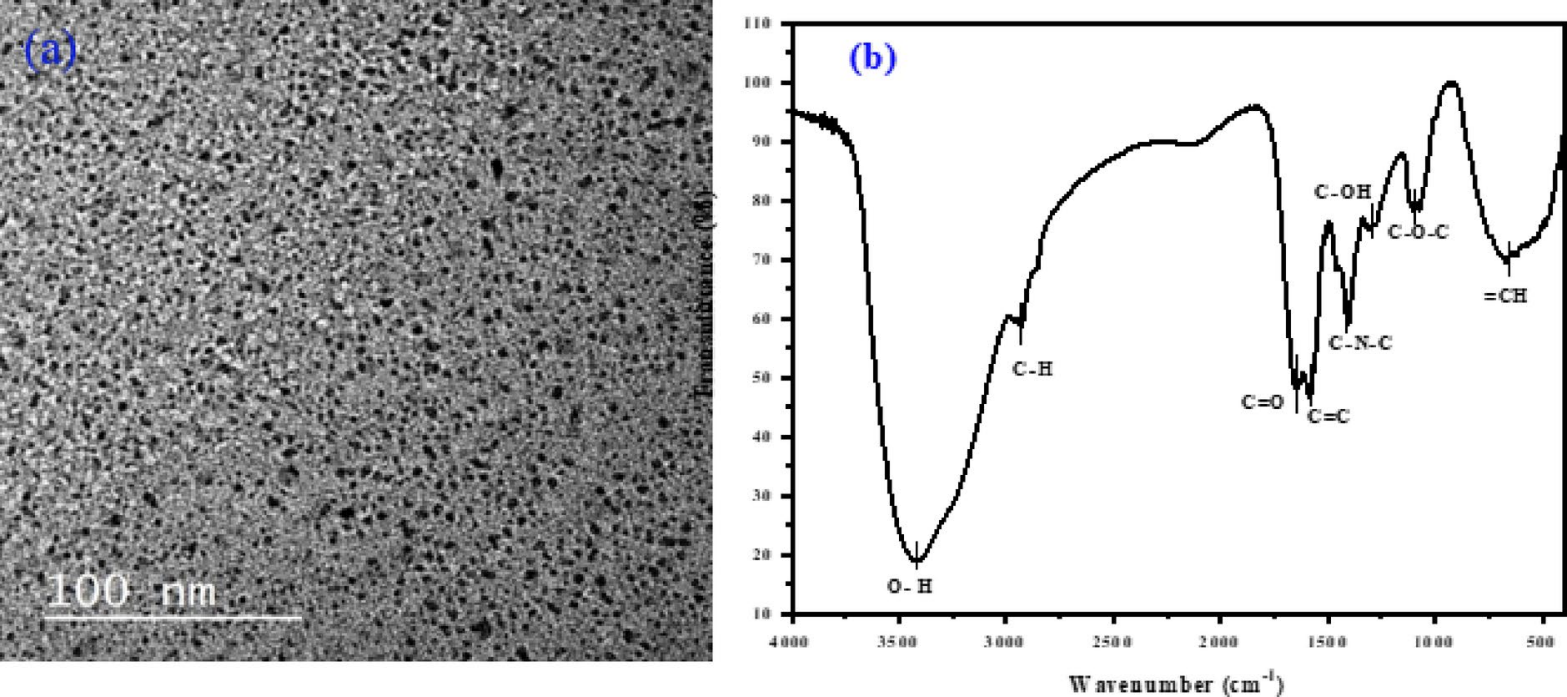
(**a**) TEM image at 100 nm (**b**) FTIR spectra of CQDs.

The CQDs’ morphology was described using TEM in Fig. 6a. TEM exhibits a uniform semi-spherical shape, almost no clear aggregations, a mean particle size of approximately 2.88 ± 0.91 nm, a narrow size distribution of 1.13–6.26 nm, and a good dispersion. The surface of CQDs was thought to contain a great deal of functionality, and FTIR spectroscopy was used to characterize these functions. As shown in Fig. 6b, owing to the stretching vibration of the O–H group, the peak at 3418 cm⁻¹ becomes explained^10,22^ the O–H bond to the carbon surface and the water absorbed on the product surfaces is connected to that. The stretching vibration of C–H is responsible for the peak at 2921 cm^− 1^. The vibrational absorption of C=O and C=C is associated with the peaks at 1650 cm^− 1^ and 1568 cm^− 1^^37^, respectively. Moreover, the bending vibrations of the C–N–C bond can be linked to the band at 1412 cm^− 1^, the peaks located at 1297, 1093, and 650 cm^− 1^ indicated C–OH, C–O–C are present and =CH respectively^22^. It is evident that numerous amino and hydroxyl groups are covering the dots surface. These functional groups on the CQD surface are what give them such good aqueous media dispersion^15^. CQDs’ water-soluble properties are explained by the presence of oxygen-containing functional groups on their surface, specifically carboxyl (–COOH) and hydroxyl (–OH). They may produce long-lasting colloids in polar solvents or water, which help with their solubility and gives them fluorescence properties^40^.

#### XPS analysis

**Fig. 7:**
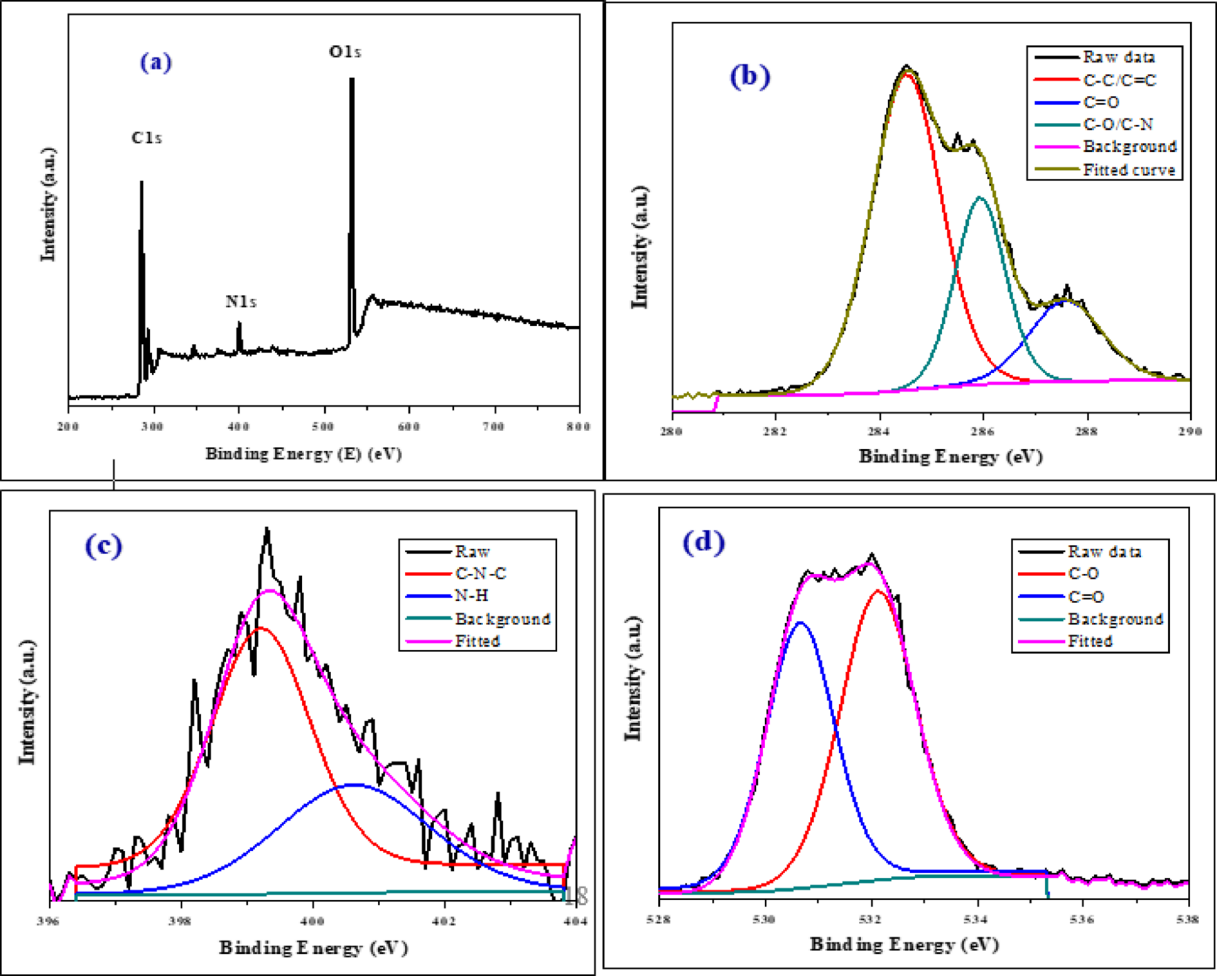
XPS spectra (**a**) survey, (**b**) high-resolution C scan, (**c**) high-resolution N scan, and (**d**) high-resolution O scan for CQDs surface.

XPS is a nondestructive technique for analyzing surface chemistry by measuring the energy distribution of photoelectrons excited by monochromatic photons. Using X-ray photoelectron spectroscopy (XPS), the CQDs were analyzed to obtain further understanding of the structural information. Carbon, nitrogen, and oxygen 1s peak values are responsible for the major peaks at 285.17, 399.9, and 531.71 eV, respectively^10^ shown in Fig. 7a, and the atomic concentrations were 60.04, 6.96 and 31.35%, respectively. The C1s spectrum of CQDs, as displayed in Fig. 7b, identified three peaks associated with C–C/C=C, C–N/C–OH, and C=O at 284.51, 285.93, and 287.58 eV^2,41^. The N1s spectra are displayed in Fig. 7c, N–C–N and N–H may be represented by the two peaks at 399.21 and 400.61 eV, respectively^37,42^ which are characteristic peaks of pyridinic nitrogen^10^ and pyrrolic nitrogen^43^ respectively. These results confirm the presence of both pyridinic and pyrrolic nitrogen in our CQDs. Figure 7d displays the O1s spectra of CQDs; the C=O and C–OH/C–O–C groups are responsible for the two prominent peaks at 530.66 and 532.12 eV^7^, Show Table 2. The high carbon content (60.04%) in artichoke leaves constitutes the foundational structure of the CQDs, which are likely composed of both sp^2^-hybridized graphitic domains and sp^3^-hybridized diamond-like regions^15^. Additionally, artichoke leaves are abundant in phenolic compounds, including caffeic acid derivatives and flavonoids such as luteolin and apigenin^27,28^. These compounds can incorporate oxygen-rich functional groups throughout the synthesis process. The substantial oxygen content (31.35%) suggests a high level of surface functionalization with groups like hydroxyl (–OH), carboxyl (–COOH), and epoxy (–C–O–C). The presence of these oxygen-containing groups improves hydrophilicity, biocompatibility, and optical properties^40^. Also, organic compounds and phenolic substances in artichoke leaves may affect nitrogen doping. Although not nitrogen-rich, their nitrogen levels can improve electronic properties and fluorescence efficiency. CQDs likely have a core-shell structure, with a graphitic carbon core and a shell featuring various oxygen and nitrogen functional groups that impact surface properties^40^. This composition makes CQDs promising for applications needing high biocompatibility and optical activity. The results of the FTIR study and XPS concurred; thus, it suggested that the as-synthesized CQDs’ surface had several hydrophilic and hydroxyl groups. XPS also measures the relative amounts of sp^2^ and sp^2^ hybridized carbon atoms. The calculated sp²/sp³ ratio of CQDs was 2.34; this suggests that the material has undergone a degree of graphitization, resulting in a structure dominated by planar hexagonal networks characteristic of sp^2^-hybridized carbon.

**Table 2:** The binding energy values and peak areas (or atomic percentages) for each fitted peak in the C1s, N1s, and O1s spectra.

Name	Peak BE	FWHM eV	Area (*P*) CPS.eV	Atomic %
C1s	284.51	1.58	13,936.77	59.04
C1s Scan A	287.58	1.65	3717.17	15.77
C1s Scan B	285.93	1.14	5942.57	25.19
N1s	399.21	1.76	1743.33	59.06
N1s Scan A	400.61	2.72	1207.63	40.94
O1s	532.12	1.68	14,698.66	54.83
O1s Scan A	530.66	1.52	12,119.17	45.17

**Fig. 8:**
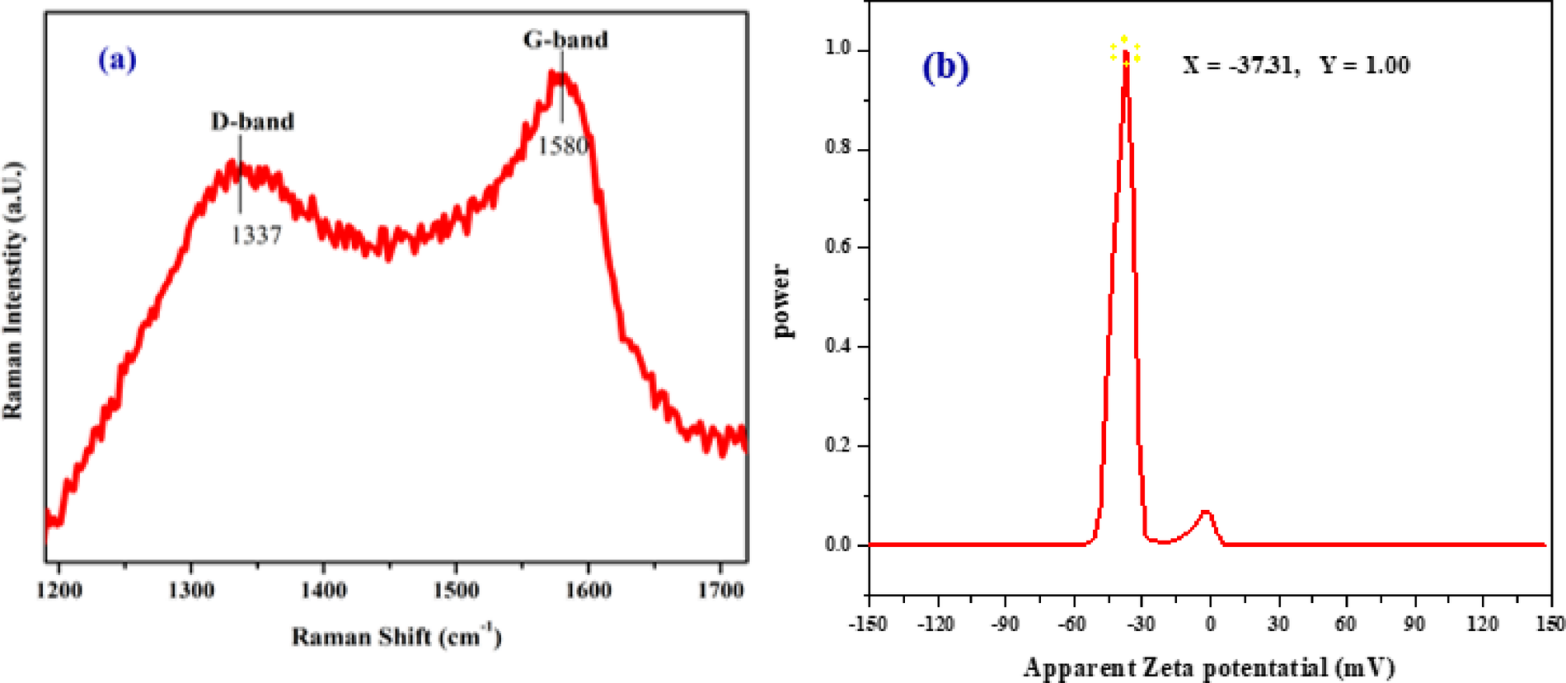
(**a**) Raman spectrum; (**b**) Zeta spectrum of CQDs.

Moreover, Raman measurements have been conducted on the synthesized CQDs, and the results are shown in Fig. 8a. The lower-power D-band at 1337 cm^−1^ and the G-band at 1580 cm^−1^ are the two peaks that can be detected. Graphitic character of the carbon framework (C–C in-plane vibrations of sp^2^-hybridized bonded carbons) is associated with the crystalline G-band, whereas the disordered D-band is associated with defects in the graphite lattice (sp^3^ hybridized carbon networks)^1,3,10,15^. The ratio (I_D_/I_G_) of the D-band to G-band intensities is frequently used to show the amount of graphitization and the concentration of defects in the graphitic structure of a carbon material that resembles graphene^1,15,44^, a greater G-band to D-band intensity ratio indicates a higher degree of graphitization and better crystalline^3^ and vice versa. The I_D_/I_G_ ratio of the synthesized CQDs was calculated to be 0.48 which is comparatively low and indicates CQDs with moderate-to-low disorder, thus indicating a reasonable degree of graphitization in (line with XRD result) Thus, it can be concluded that there are fewer defects in the sp² lattice compared to highly disordered carbons, which demonstrated the CQDs are crystallized in the graphitic structure^10^. It also suggests that the graphitic shells have a high enough integrity to effectively prevent oxidation and corrosion of the core material^1^. Compared to other CQDs or carbon nanomaterials, our CQDs are most likely to have a high degree of graphitization, which can enhance properties like electrical conductivity and stability^3,12^. The correlation between Raman spectroscopy findings (I_D_/I_G_ ratio) and XPS data (sp^2^/sp^2^ carbon ratio) provides insights into the structural composition and graphitization of carbon-based materials. Thus, we can conclude that the observed relationship between the I_D_/I_G_ and sp^2^/sp^2^ ratios is consistent with known patterns in carbon-based materials, which generally show larger, more ordered graphitic domains as the sp^2^ content rises. This is reflected in a lower I_D_/I_G_ ratio because of less structural disorder^3^. The water-soluble stability of CQDs was measured using the zeta potential, and the zeta spectrum is presented in Fig. 8b. Greater electrostatic repulsion between neighboring particles in dispersion is indicated by a greater zeta potential value, which also indicates excellent colloidal stability and high resistance to agglomeration^21^. A value of ± 30 mV indicates physical stability, and a value of ± 60 mV indicates total stability. For our CQDs, the zeta potential value of − 37.31 mV indicated that the synthetic CQDs are in an excellent colloid state and showed that the surface of the dots is negatively charged.

#### Optical characteristics

**Fig. 9:**
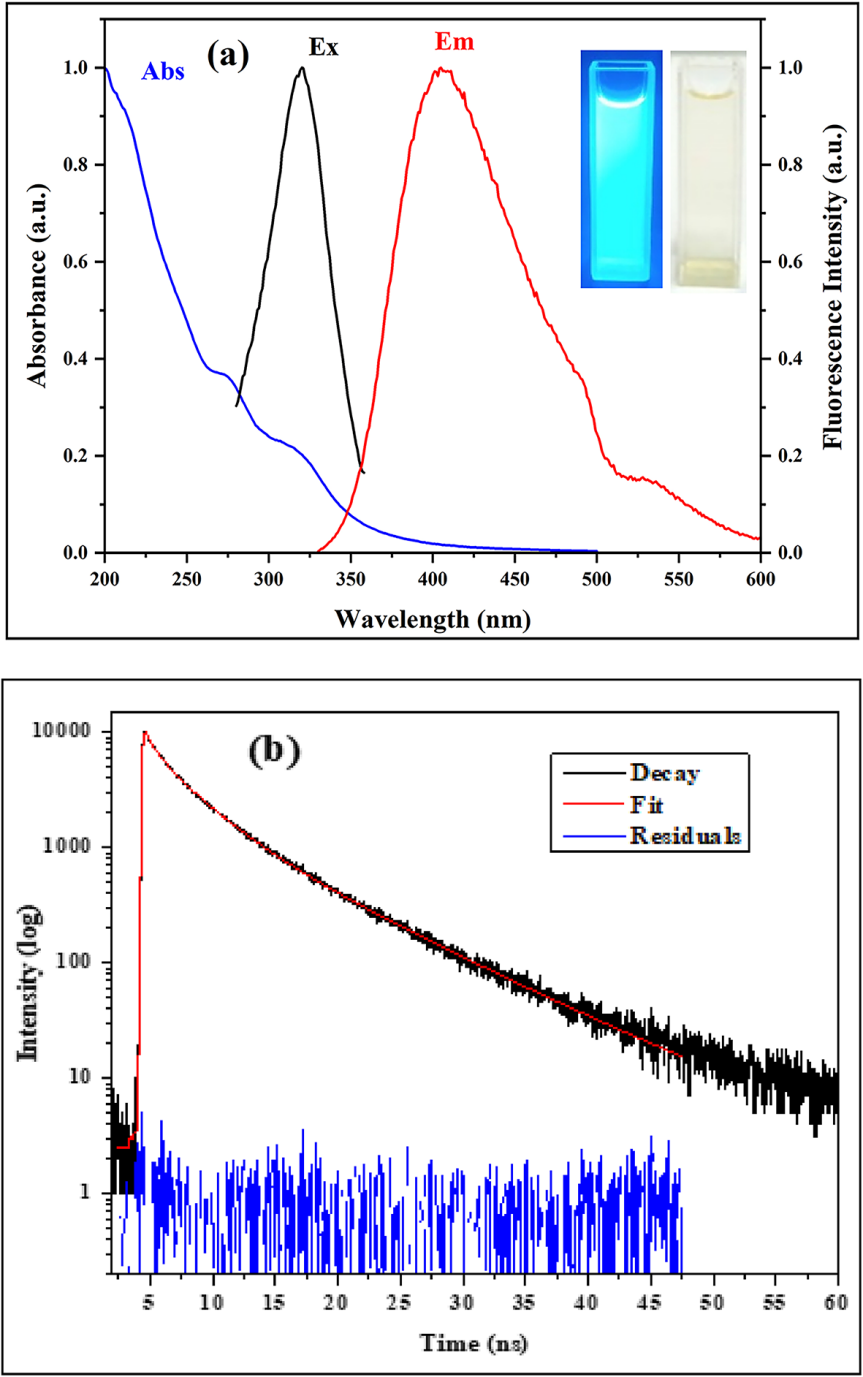
(**a**) CQDs’ UV–Vis absorption, excitation, and emission spectra. The inset shows photographs of CQDs solutions under visible light (right) and UV illumination at 365 nm (left). (**b**) CQDs’ fluorescence lifetime upon excitation at 320 nm. The black line represents experimental data, the red line is the fitting, and the blue line is the residual.

As shown in the inset of Fig. 9a the photo of CQDs solutions showed a faint yellow tint in the visible light and a brilliant blue fluorescence when exposed to UV light at 365 nm. The band gap of synthesized CQDs was calculated from UV-Vis spectra and was found to be 4.26 eV^10^. Observations reveal that when CQDs are excited at 320 nm, their photoluminescence spectrum has a high emission peak centered at 398 nm (Fig. 9a). Furthermore, the dots’ fluorescence was observed to decay, yielding an average fluorophore lifetime of 5.4 ns as shown in Fig. 9b.

#### Cytotoxicity, antimicrobial and mICTest results

**Fig. 10:**
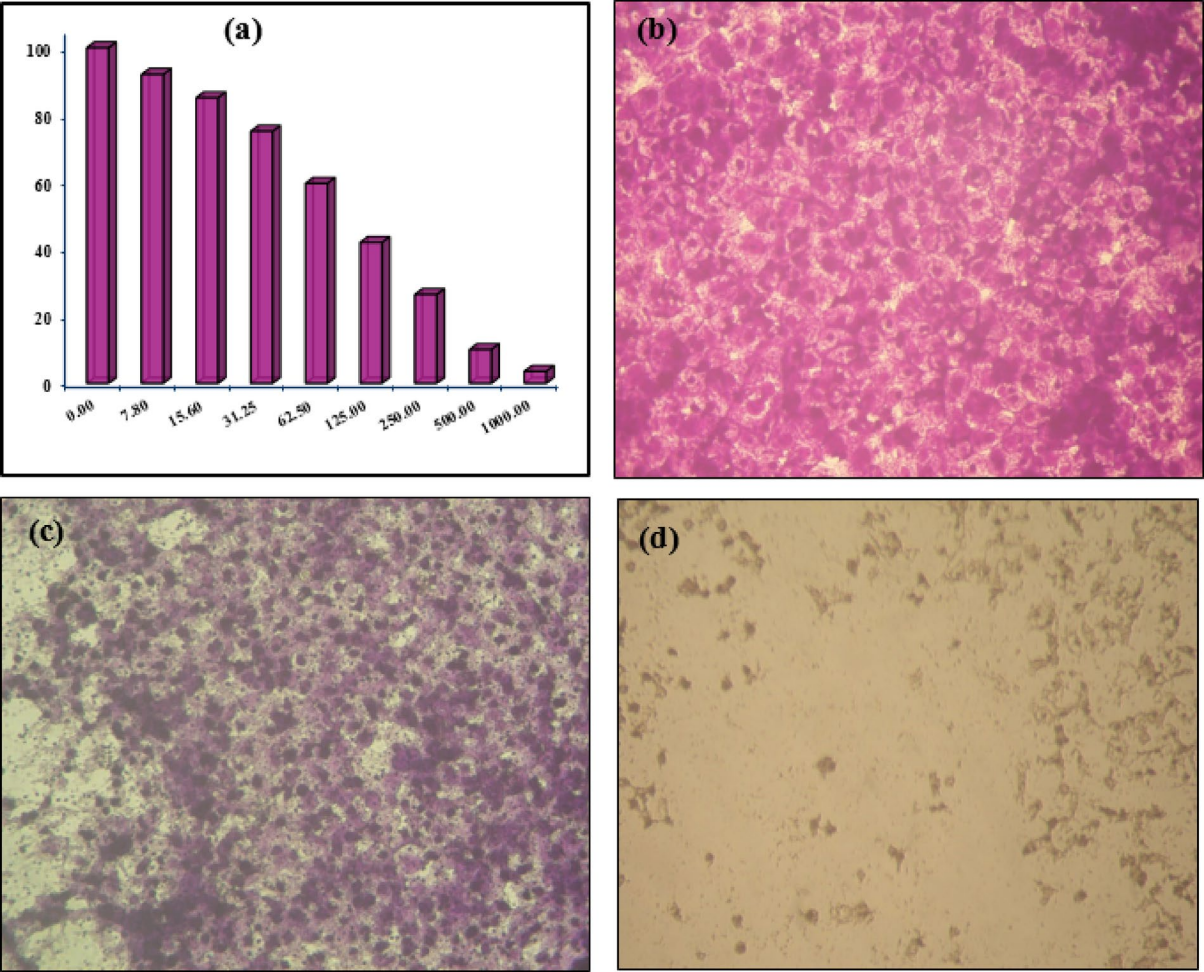
(**a**) Cell viability percentage based on CQDs concentrations; (**b**) MCF-7 control cells (0 µg/mL); (**c**) treated with 100 µg/mL; and (**d**) 1000 µg/mL CQDs. The cytopathic effects were microscopically observed at ×200.

To evaluate the cytotoxic effect of CQDs, the viability of the MCF-7 (HTB-22) cell line exposed to CQDs was assessed using the MTT assay. The relationship between the number of surviving cells and the concentration of CQDs is illustrated to create the survival curve for the MCF-7 cell line following treatment with CQDs. In Fig. 10, we observed the effects of different doses of CQDs on MCF-7 cells over a 48-h incubation period. The results indicated that at concentrations nearing 1000 µg/mL, there was a significant reduction in cell viability, with over 95% of the MCF-7 cells experiencing cell death. Furthermore, the IC_50_ value for these CQDs has been determined to be 96.5 ± 5.3 µg/mL, highlighting their effectiveness in inhibiting cell growth in line with previous research^45,46^ although it can be low potential comparatively to some studies and within the range according to another^47^. The strong colloidal stability provided by a zeta potential of − 37.31 mV ensures consistent nanoparticle behavior in biological environments, improving their likelihood of interacting with target cells like MCF-7. Additionally, such nanoparticles may exhibit reduced nonspecific uptake by normal cells, enhancing their selectivity for cancer cells. This data provides valuable insight into the potential application of CQDs in cancer treatment. The cellular morphology was observed using an inverted microscope (CKX41; Olympus, Japan) equipped with a digital microscopy camera.

**Fig. 11:**
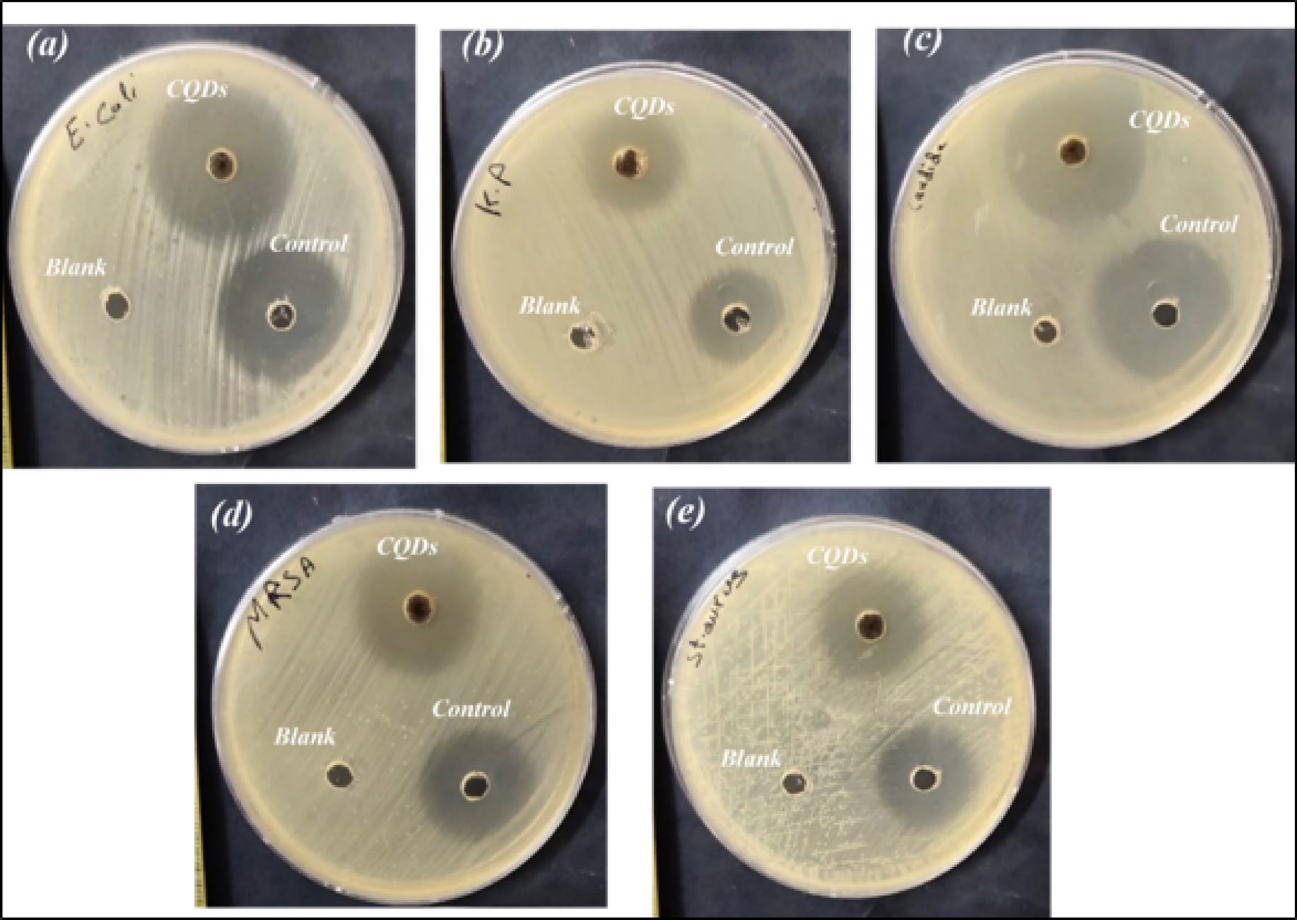
Photographs of culture dishes containing (**a**) *E. coli*, (**b**) *K. pneumoniae*, (**c**) *C. albicans*, (**d**) *MRSA*, and (**e**) *S. aureus* treated with the prepared CQDs. Blank refers to the solvent, and control refers to commercial antibiotics.

**Fig. 12:**
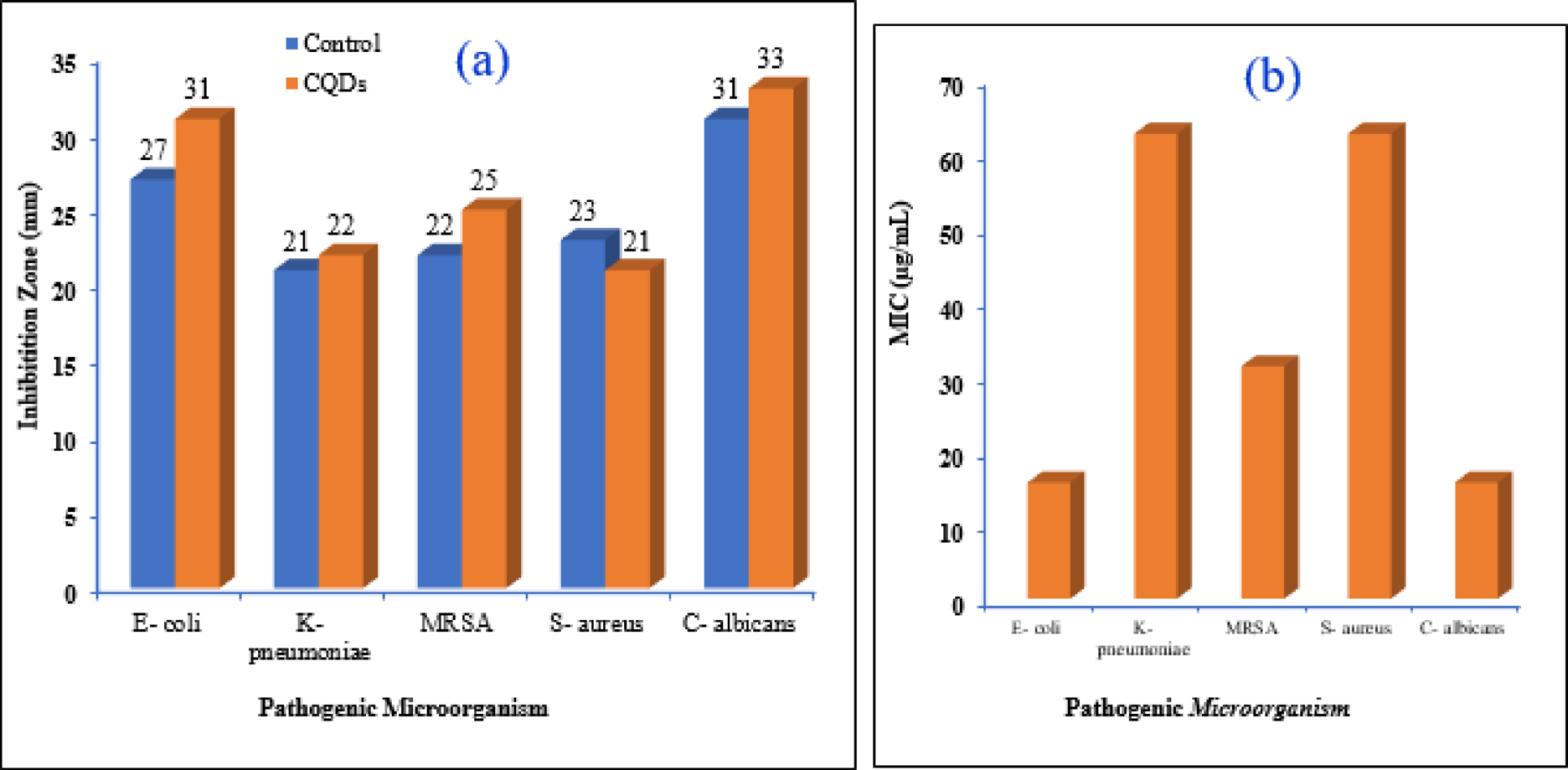
Effects of CQDs in inhibiting various microorganisms: (**a**) inhibition zones and (**b**) minimum inhibitory concentration.

**Scheme 4:**
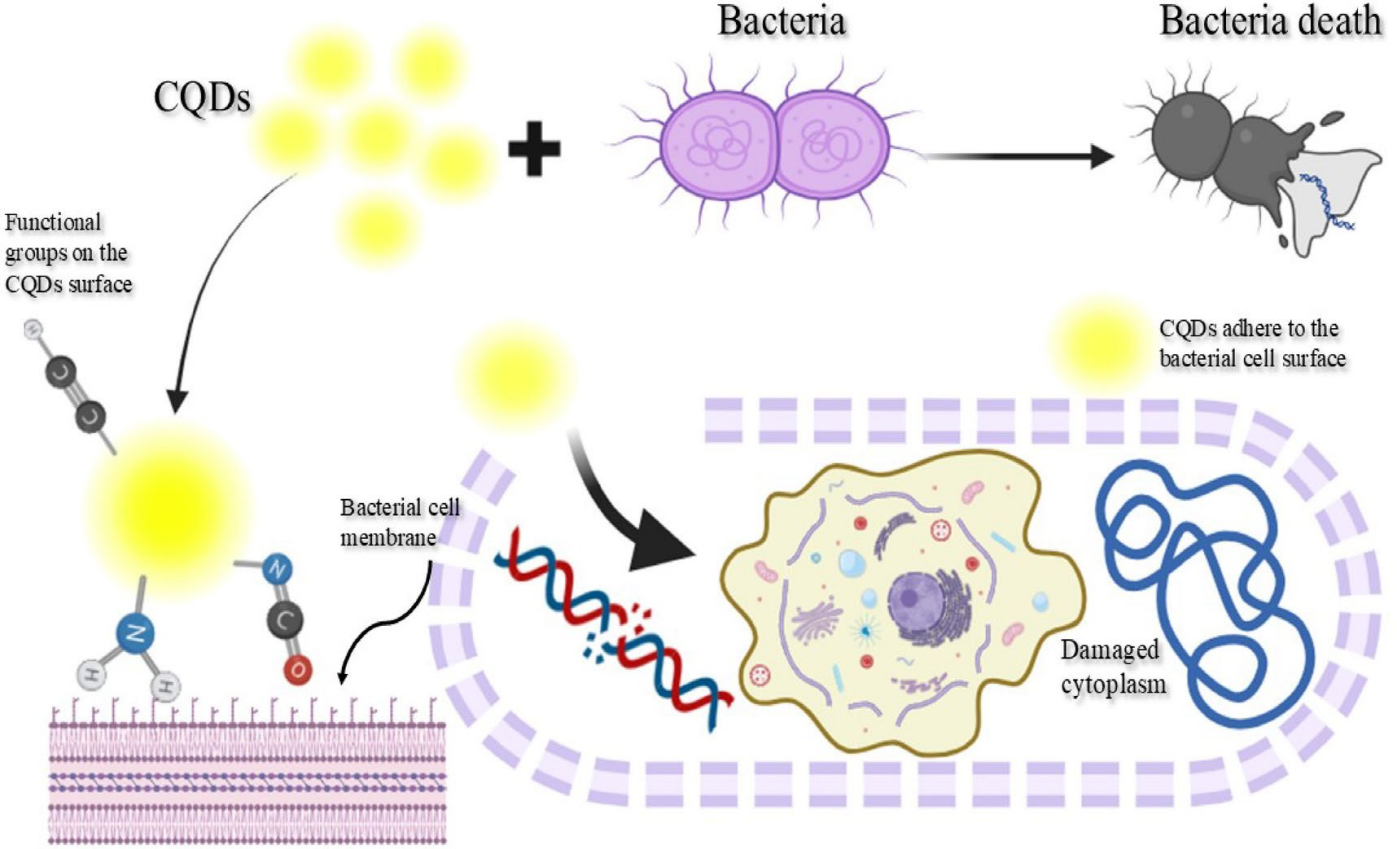
Interaction of CQDs with bacteria in antimicrobial activity test.

Effectively inhibiting microbial pathogens in the treatment of infectious diseases is a significant issue. So, the CQDs underwent testing to determine their antimicrobial properties against both Gram-positive and Gram-negative bacterial pathogens, as well as a potential human fungus pathogen under in vitro circumstances. The results indicate the inhibition zone (in mm) of the CQDs against the examined strains of bacteria and fungi, which were then compared with Ciprofloxacin (a fluoroquinolone antibiotic drug used to treat bacterial infections) and Fluconazole (a long-acting antifungal medication) as typical reference medications. The CQDs showed varying levels of inhibition against different bacterial strains, The diameter of inhibition zones and MIC values were summarized in Figs. 11 and 12; Table 3. As illustrated, the activity was higher than the control for *E. coli*,* K. pneumoniae*,* MRSA*, and the fungus *C. albicans* by 14.8%, 4.76%, 13.63%, and 6.45%, respectively. Only *S. aureus* showed a lower effect, which was only 8.69% lower than the control. The antimicrobial activity of CQDs prepared from artichoke leaves may be linked to the existence of functional groups and phenolic substances and their ability to bind to bacterial cell walls, thereby inhibiting microbial growth^48^. Because carboxyl (–COOH) groups can act as electron donors or acceptors, they can increase the generation of ROS, which is essential for destroying microbial proteins, lipids, and DNA^49^. The process of hydrogen bonding promotes adherence to microbial surfaces and permits physical membrane rupture. Additionally, these groups can produce hydroxyl radicals, which can lead to oxidative stress in microorganisms. Like carboxyl groups, hydroxyl (–OH) groups enhance physical interactions by encouraging adherence to microbial surfaces. Also, the characteristics of nanoparticles cause the bacterial membrane to rupture due to their vast surface area that can adhere firmly to the bacterial cell surface and quickly pass through the membrane as shown in Scheme 4. As a result, intracellular components of the cell would flow out and kill the bacterial cell^50^. Because of the widespread usage of antibacterial compounds in all facets of daily life, including water purification, health and medical supplies, and preservation supplies, and the CQDs showed excellent antimicrobial activity; thus, these CQDs may be used as an antibacterial agent with reasonable safety margins to inhibit bacterial growth. Because of this, they are especially appealing for medicinal applications where antibacterial action is required^36^.

The lower the MIC value, the more effective the antimicrobial agent against microorganisms. Minimum inhibitory concentration values (MIC, µg/mL) of the tested CQDs proved that it is a good inhibitor against *E. coli* and *Candida* as they show the lowest MIC values (15.62 µg/mL), indicating that CQDs are particularly effective against these pathogens^35^. The CQDs demonstrate a higher effectiveness against *MRSA* than *S. aureus*, as indicated by the lower MIC for *MRSA* (31.25 µg/mL).

This suggests that the CQDs may have a specific mechanism that targets *MRSA* effectively, which is significant given the challenges associated with treating *MRSA* infections. The MIC for *K. pneumoniae* is the same as that for *S. aureus* (62.5 µg/mL), suggesting that CQDs have moderate effectiveness against these Gram-positive and Gram-negative microorganisms, respectively. The MIC for *Candida* is also low (15.62 µg/mL), indicating that CQDs can be effective antifungal agents, which is particularly relevant in the context of rising antifungal resistance. Based on these findings, it was discovered that CQDs showed good antimicrobial activity against Gram-positive and Gram-negative bacteria^51^. Thus, the studies highlighted the significance of these substances’ chemical structures, especially the existence of delocalized electron systems and free phenolic hydroxyl groups that enable proton exchange. The antibacterial activity of these compounds is attributed to their aromatic ring structure, and the precise location of hydroxyl groups plays a crucial role in this regard^32^. Additionally, CQDs’ high fluorescence performance, non-toxicity, capacity to be functionalized for targeted therapies, and ability to emit in the near-infrared (NIR) region, which is beneficial for deep tissue imaging, make them attractive for in vivo uses in drug delivery, bioimaging, antimicrobial therapy, and cancer treatment^52^. Also, our synthesized CQDs are promising candidates for drug delivery due to their biocompatibility. Surface modifications such as targeting ligands, PEGylation, and pH-responsive linkages are crucial for enhancing specificity, circulation time, and controlled drug release^53^. The role of some CQDs in bioactive applications is summarized in Table 4.

**Table 3:** The values of the inhibitory zone diameter ± sd for four different bacterial species and *C. albicans* fungus following CQDs therapy, antibiotic samples, and the MIC values for the prepared CQDs.

Pathogenic microorganisms	Inhibition zone diameter (mm)		MIC (µg/mL)
	Control (Antibiotic)	CQDs	
*Escherichia coli (ATCC 8739)*	27 ± 0.1	31 ± 0.2	15.62 ± 1.6
*Klebsiella pneumoniae (ATCC 13883)*	21 ± 0.1	22 ± 0.1	62.50 ± 5.8
*Methicillin-resistant Staphylococcus aureus (ATCC 33591) (MRSA)*	22 ± 0.2	25 ± 0.2	31.25 ± 3.1
*Staphylococcus aureus (ATCC 6538)*	23 ± 0.1	21 ± 0.1	62.50 ± 6.2
*Candida albicans (ATCC 10221)*	31 ± 0.1	33 ± 0.1	15.62 ± 2.0

**Table 4:** The role of some CQDs in bioactivity applications.

Source	Size	Bioactivity	References
m-Aminophenol and phosphoric acid	2.75 nm to 4.25 nm	Antibacterial activity against *Escherichia coli* (*E. coli*) and *Staphylococcus aureus* (*S. aureus*)	^54^
5-Fluorouracil citric acid and urea	4.82 ± 0.75 nm	Cytotoxicity against normal human lung fibroblast (GM07492A) and human breast cancer (MCF-7) cell lines	^55^
Folic acid	2.69 ± 0.51 nm	Cytotoxicity of free MTX, CQDs, Ex, MTX-CQDs, and Ex@MTX-CQDs against MCF-7 cells	^45^
Citric acid, l-glutathione and polyethylene polyamine (PEPA)	2.5 nm	In vitro cytotoxicity against MC3T3-E1 cells and In vitro antimicrobial against *S. aureus*, MRSA, *L. monocytogenes*, *E. faecalis*, *E. coli*, *S. marcescens*, *P. aeruginosa*	^33^
Artichoke leaves	2.88 nm	Cytotoxicity against MCF-7 In vitro antimicrobial activity against (Methicillin-resistant *Staphylococcus aureus* (MRSA), *Staphylococcus aureus*, *Klebsiella pneumoniae*, *Escherichia coli* and *Candida albicans*	This work

#### Fluorescence quantum yield

The PL quantum yield (QY) was found to be 3.32 ± 0.046%. Although this value may be considered low compared to other CQDs, particularly those synthesized by chemical precursors^44^, yet they are within the range reported for many biowaste-derived CQDs^56^. Depending on the intended application, a QY of 3.32 ± 0.046% mght be sufficient. For instance, in biomedical imaging or sensing applications where high sensitivity is not critical, such QYs can still provide useful fluorescence properties. It is essential to realize that the carbon dots’ surface structure and any potential modifications made during the manufacturing process have an impact on the quantum yield value^44^. Also, the PL quantum yield can be enhanced by surface passivation by functional groups and co-doping of heteroatoms^38,40,57^.

#### Stability of CQDs

##### Photostability of CQDs

**Fig. 13:**
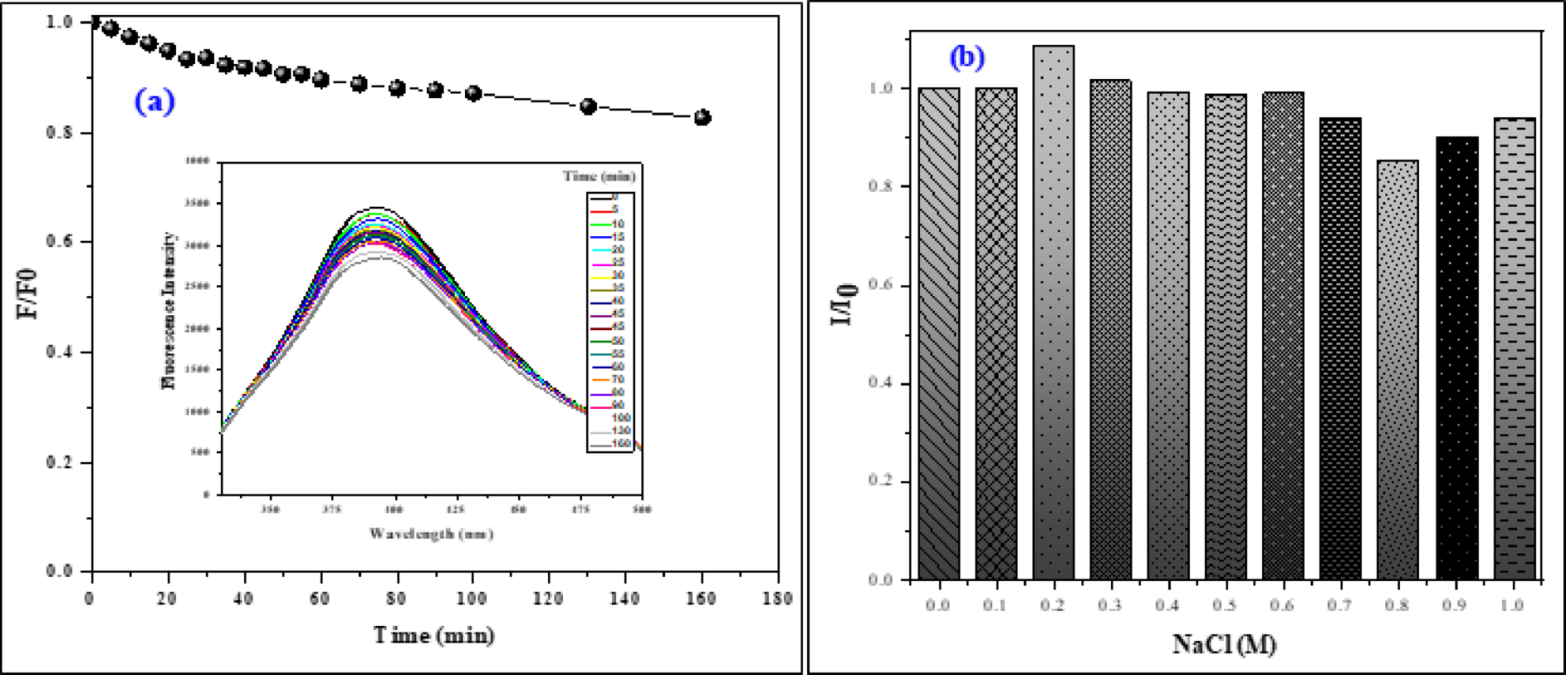
(**a**) Fluorescence spectra of CQDs under continuous UV from 0 min to 160 min. (**b**) The impact of NaCl concentration on the fluorescence intensity of the CQDs aqueous solution at 320 nm. I₀ is the CQDs dispersion’s PL intensity without NaCl, whereas I is the PL intensity at diverse concentrations of NaCl.

We are aware of the importance of CQDs’ photostability for cell bioimaging and investigated how CQDs emit light when exposed to continuous 320 nm UV light for 160 min. Our observations in Fig. 13a revealed that the CQDs do not undergo photobleaching, while the emission intensity decreased by only 18.26% even after 160 min of continuous UV light exposure. This suggests that the CQDs exhibit excellent photostability^23^. After 4 h of exposure, PL loss was observed, and after 150 h, it stabilized at 10% of its initial intensity, according to some research. Another argument is that even after 80 min of exposure, there was no appreciable decrease in fluorescence intensity. According to some, the produced CDs maintained 92% of their original fluorescence intensity even after being exposed to radiation for 120 min. Another study mentioned that the fluorescence intensity of N-CDs did not significantly alter when the testing duration was extended by thirty minutes. After being exposed to UV lamps for 180 min, 96% of the initial intensity was still present^58^. From this, we decisively deduced that the synthesized CQDs fall within the accepted range.

##### Effect of ionic strength

How NaCl affects CQDs’ fluorescence properties was also investigated under 320 nm. NaCl was tested from 0.1 M to 1.0 M, and CQDs’ fluorescence intensity was assessed at various NaCl concentrations. As shown in Fig. 13b, no matter how high or low the concentration, PL intensity is rarely floating. No clear decrease in intensity is noted up to the concentration of NaCl (0.7 M). A decline of less than 7% was identified even at elevated NaCl concentrations (1.0 M), proving the CQDs’ good fluorescence stability in the electrolyte. Moreover, adding NaCl in a concentration of 0.2 M increases the fluorescence intensity by 9%, which implies that the synthesized CQDs have excellent stability in a high ionic strength environment^12^ providing an advantageous platform for applications related to sensing and imaging^11^.

#### Sensing of hydrogen peroxide

**Fig. 14:**
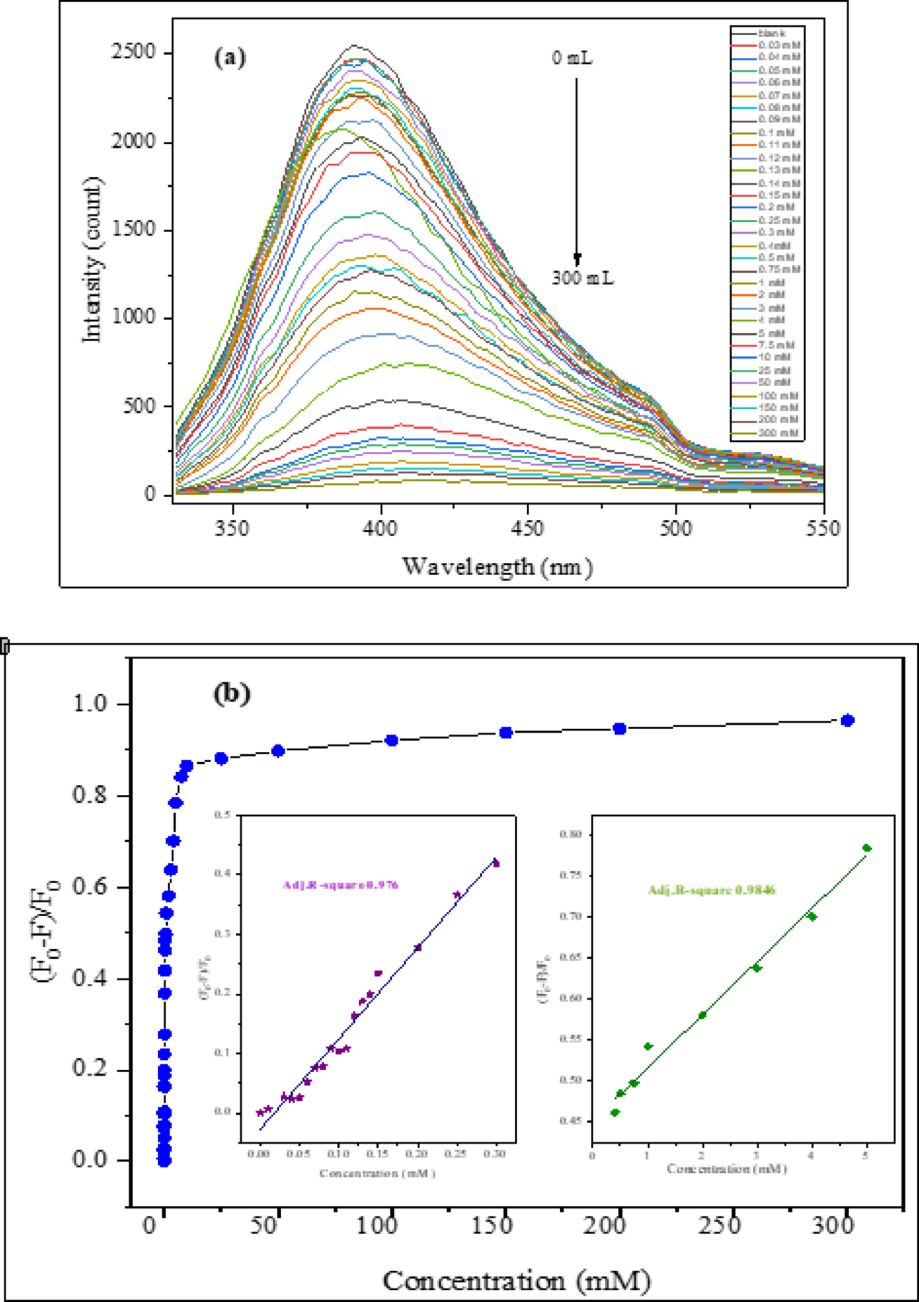
Application of CQDs for H_2_O_2_. (**a**) Influence of H_2_O_2_ on CQD PL emission. The addition of H_2_O_2_ causes a decrease in PL intensity. (**b**) Relative intensity plotted linearly with H_2_O_2_ concentration.

The detection sensitivity of hydrogen peroxide (H_2_O_2_) was examined to ensure the practicality of CQDs as a biosensing tool. Figure 14 demonstrates how the introduction of H_2_O_2_ quenched the photoluminescence (PL) emission of the synthesized CQDs. As shown in Fig. 14a as H_2_O_2_ was added sequentially (0–0.3 M), the PL emission of CQDs progressively decreased, which can be attributed to the interaction between H_2_O_2_ and CQDs. With an R² value of 0.976 from 0 to 0.3 mM and 0.984 from 0.4 to 5 mM, the PL quenching efficiency of CQDs shows a linear correlation, as depicted in Fig. 14b. Using the formula LOD = 3σ/m (where σ and m represent the standard deviations and slope of the linear calibration curve), the limit of detection (LOD) for H_2_O_2_ was calculated at 0.112 mM. Given that H₂O₂ is an oxidizing agent, we expect this reduction in CQDs may lead to the formation of non-radiative recombination centers or the destruction of emissive traps by oxidizing the surface functional groups of CQDs, which are typically responsible for their PL. Additionally, H_2_O_2_ can act as a surface quencher, decreasing PL intensity and facilitating non-radiative decay.

## Conclusion

As an effective waste management strategy, we utilized artichoke leaves as a precursor for the preparation of carbon quantum dots (CQDs) through a hydrothermal method. The optimal duration for producing CQDs was found to be 18 h (S3) at 240 °C. The synthesized CQDs demonstrate an acceptable quantum yield (QY) of 3.32 ± 0.046%, a long fluorophore lifetime of 5.4 ns, and remarkable stability in environments with high ionic strength or under continuous UV radiation, along with a limit of detection (LOD) of 0.112 mM for peroxide sensing. Furthermore, this study explored the impressive antibacterial and anticancer properties of carbon quantum dots derived from artichoke leaves, underscoring their potential applications in the medical field.

## Electronic supplementary material

Below is the link to the electronic supplementary material.

Supplementary Material 1

## Data Availability

All informations produced or examined throughout the research have been included in this published article.
