# Supplementary material for: In vitro antimicrobial and anticancer potentials of green synthesized luminescent carbon quantum dots derived from artichoke leaves
Source: Sci Rep. 2025 May 9;15:16199. doi: 10.1038/s41598-025-99841-9 (PMC12064798; doi:10.1038/s41598-025-99841-9)
Supplement: Supplementary file 1 — Supplementary Material 1 [file 41598_2025_99841_MOESM1_ESM.pdf]

Statistical Analysis of "*In vitro* Antimicrobial and Anticancer Potentials of Green Synthesized Luminescent Carbon Quantum Dots Derived from Artichoke Leaves" Manuscript

Table 1 Minimum inhibitory and bactericidal/fungicidal concentrations (MIC and MBC) of CQDs against bacterial and fungal pathogens strains

Pathogenic Microorganism	MIC of CQDs (µg/mL)	MBC (µg/mL)
Staph. aureus (ATCC 6538)	62.5 ± 6.2^{a}	125 ± 12.5 ^{a}
Methicillin-resistant *S. aureus* (*ATCC 33591)*	31.25 ± 3.1^{b}	62.5 ± 6.3 ^{b}
Escherichia coli (ATCC 8739)	15.62 ± 1.6 ^{c}	15.62 ± 1.8 ^{c}
Klebsiella pneumoniae (ATCC 13883)	62.5 ± 5.8^{a}	125 ± 15.0 ^{a}
Candida albicans (ATCC 10221)	15.62 ± 2.0 ^{c}	62.5 ± 7.0 ^{b}
F-value	95.46	69.66
p-value	< 0.001	< 0.001

Values are presented as mean ± standard deviation. Different superscript letters (a, b, c) within the same column indicate statistically significant differences between means at $p < 0.05$ (ANOVA followed by Tukey post hoc test). F-value and p-value are statistics calculated in a one way ANOVA (Analysis of Variance) test.

Comment on results

The sample demonstrated varying degrees of antimicrobial activity against the tested bacterial and fungal strains, as reflected in both **MIC** (Minimum Inhibitory Concentration) and **MBC** (Minimum Bactericidal/Fungicidal Concentration) values.

1. Highest Sensitivity (Lowest MIC/MBC):

- ***Escherichia coli* (ATCC 8739**) exhibited the **highest sensitivity** to the sample, with both MIC and MBC values at **15.62 µg/mL**. This suggests the sample has strong bacteriostatic and bactericidal activity against *E. coli*.

- ***Candida albicans*** also showed good sensitivity (MIC = 15.62 µg/mL), though its MBC was higher (62.5 µg/mL), indicating a stronger inhibitory than fungicidal effect.

2. Intermediate Sensitivity:

- Methicillin-resistant Staphylococcus aureus (MRSA) showed moderate sensitivity (MIC = 31.25 µg/mL, MBC = 62.5 µg/mL), suggesting the sample could be effective even against drug-resistant strains.

3. Lower Sensitivity (Highest MIC/MBC):

- *Staphylococcus aureus* and *Klebsiella pneumoniae* both had MICs of 62.5 µg/mL and MBCs of 125 µg/mL, indicating lower susceptibility and suggesting that higher concentrations of the sample are needed to inhibit or kill these organisms.

Statistical Significance: The high **F-values** (MIC: 95.46, MBC: 69.66) and **p-values < 0.001** indicate that the differences in antimicrobial susceptibility among the microorganisms are **highly statistically significant**. This confirms that the sample's effectiveness varies considerably between different microbial strains.

Table 2. Antimicrobial Activity Test

Pathogenic microorganism	Sample	Control	t-value	p-value
***Staph. aureus* (ATCC 6538)**	21 ± 0.1	23 ± 0.1	24.5	< 0.001
***Methicillin-resistant S. aureus* ATCC 33591 (MRSA)**	25 ± 0.2	22 ± 0.2	18.37	< 0.001
***Escherichia coli* (ATCC 8739)**	31 ± 0.2	27 ± 0.1	30.98	< 0.001
***K. pneumoniae* (ATCC 13883)**	22 ± 0.1	21 ± 0.1	12.25	< 0.001
***Candida albicans* (ATCC 10221)**	33 ± 0.1	31 ± 0.1	24.5	< 0.001

Values represent the diameter of inhibition zones (mm), expressed as mean ± SD. *t*-value and *p*-value are from an independent *t*-test comparing the sample and control groups. A p-value < 0.001 means the difference is highly statistically significant.

Comment on results

All tested organisms show **statistically significant differences** between the sample and control (p < 0.001). This confirms that the sample exhibits **true antimicrobial activity**

- The sample was more effective than the control against: MRSA (25 mm vs. 22 mm), *E. coli* (31 mm vs. 27 mm), *K. pneumoniae* (22 mm vs. 21 mm), *C. albicans* (33 mm vs. 31 mm).

- The inhibition zone for *S. aureus* was slightly **smaller** in the sample (21 mm) than the control (23 mm), but this difference is still statistically significant. This suggests the control agent may be more potent against this particular strain.

Table 3. Effect of Sample (CQDs) on MCF-7 Cells

Sample conc. (μg/ml)	MCF-7	
	Viability %	Inhibitory %
0*	100 ± 0	0 ± 0
7.8	92.06 ± 1.68	7.94 ± 1.68
15.6	85.11 ± 0.28	14.89 ± 0.28
31.25	75.13 ± 0.97	24.87 ± 0.97
62.5	59.58 ± 1.34	40.42 ± 1.34
125	41.98 ± 1.67	58.02 ± 1.67
250	26.45 ± 1.4	73.55 ± 1.4
500	10.06 ± 0.66	89.94 ± 0.66
1000	3.51 ± 0.25	96.49 ± 0.25

*0 = Non-treated cell control

Table 4. Effect of Reference Drug (Cisplatin) on MCF-7 Cells

Reference conc. (μg/ml)	MCF-7	
	Viability %	Inhibitory %
0	100 ± 0	0 ± 0
0.5	56.9 ± 0.56	43.1 ± 0.56
1	48.72 ± 0.87	51.28 ± 0.87
2	43.7 ± 1.29	56.3 ± 1.29
3.9	39.6 ± 0.75	60.4 ± 0.75
7.8	34.48 ± 0.57	65.52 ± 0.57
15.6	28.61 ± 0.67	71.39 ± 0.67
31.25	21.3 ± 0.52	78.7 ± 0.52
62.5	12.43 ± 0.5	87.57 ± 0.5
125	7.5 ± 0.57	92.5 ± 0.57
250	3.88 ± 0.14	96.12 ± 0.14
500	1.55 ± 0.19	98.45 ± 0.19
1000	0.61 ± 0.09	99.39 ± 0.09

Sample	IC_{50} (μg/ml)	t-test
**CQDs**	**96.59 ± 5.3**	**t-value = 31.29**<br>**p-value < 0.001**
**Cisplatin**	**0.92 ± 0.05**	

Table 3: Effect of Sample (CQDs) on MCF-7 Cells

- The viability of cells decreases with increasing concentration of **CQDs**.
- At **1000 µg/ml**, viability is **3.51%**, with **96.49% inhibition**.
- **IC_{50} for CQDs = 96.59 ± 5.3 µg/ml**, meaning this is the concentration where cell viability is reduced by 50%.

Table 4: Effect of Reference Drug (Cisplatin) on MCF-7 Cells

- Shows a much stronger cytotoxic effect at much lower concentrations.
- At **1000 µg/ml**, viability is only **0.61%**, with **99.39% inhibition**.
- **IC_{50} for Cisplatin = 0.92 ± 0.05 µg/ml**, showing it's much more potent than CQDs.

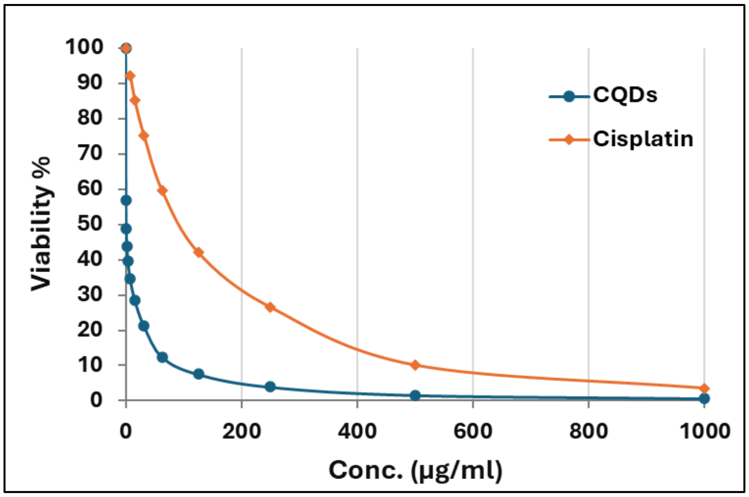

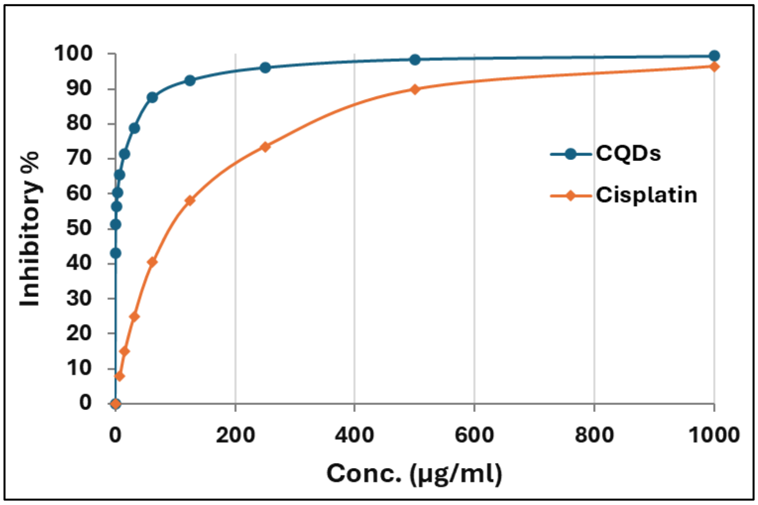

CQDs do exhibit dose-dependent cytotoxicity, but require much higher doses to reach similar levels of inhibition.

Cisplatin is significantly more effective at inhibiting MCF-7 breast cancer cells compared to CQDs.

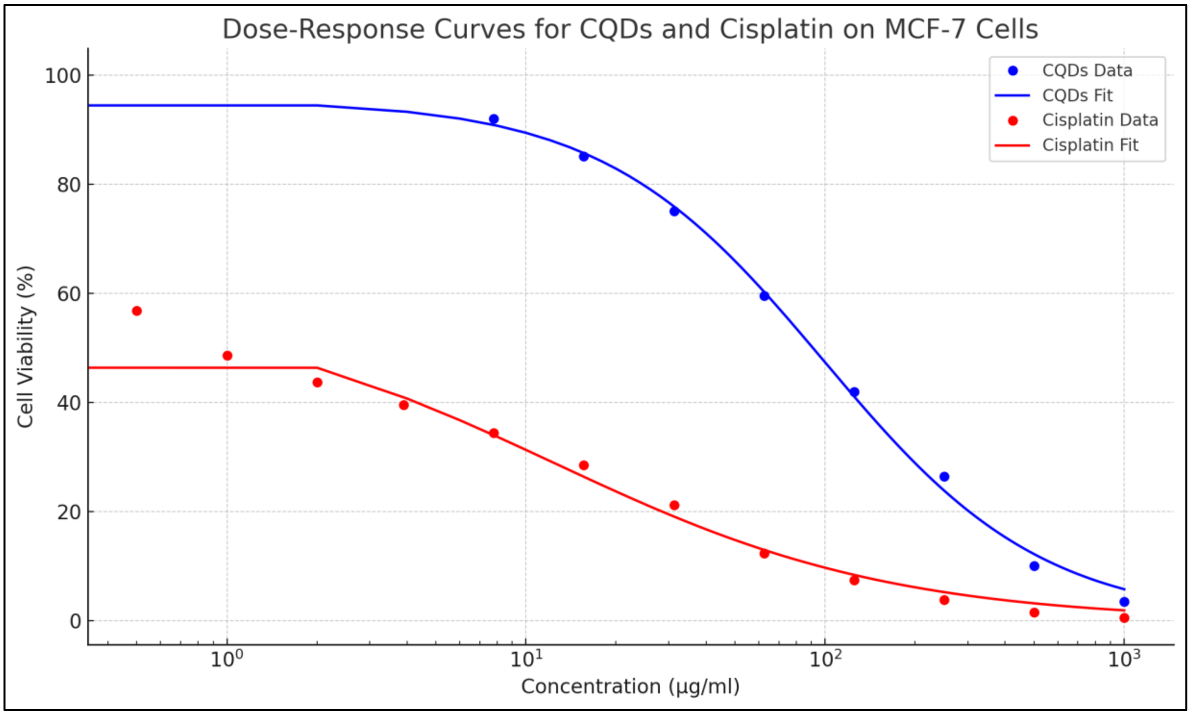

The plot above shows the **dose-response curves** of **CQDs** and **Cisplatin** on MCF-7 breast cancer cells using a four-parameter logistic (4PL) model. Here's a breakdown of the key findings.

Statistical Analysis

All experimental data were expressed as **mean ± standard deviation (SD)**. The antimicrobial efficacy of the sample was assessed using two main statistical approaches:

One-way Analysis of Variance (ANOVA) followed by **Tukey's post hoc test** was employed to compare the **minimum inhibitory concentration (MIC)** and **minimum bactericidal/fungicidal concentration (MBC)** values across different microbial strains. A significance level of **$p < 0.05$** was considered statistically significant. The presence of different superscript letters within the same column in Table 1 indicates significant differences among the tested microorganisms. F-values and p-values were reported to confirm the variation among groups.

Independent two-sample t-tests were used to compare the **diameter of inhibition zones** between the sample and control groups for each tested pathogen. All comparisons showed statistically significant differences with **$p < 0.001$**, confirming the antimicrobial activity of the sample.

For the **cytotoxicity assay on MCF-7 breast cancer cells**, viability and inhibition percentages were calculated across a range of sample concentrations. The **IC_{50} values** (concentration that inhibits 50% of cell viability) for both the test sample (CQDs) and reference drug (Cisplatin) were determined using a **four-parameter logistic (4PL) regression model**. The difference in IC_{50} values between CQDs and Cisplatin was evaluated with a **t-test**, yielding **$p < 0.001$**, indicating a statistically significant difference in cytotoxic potency.

All statistical analyses were performed using GraphPad prism V.8.3 and MS Excel 365 software.
